# The number of conspecific alarm substance donors notably influences the behavioural responses of zebrafish subjected to a traumatic stress procedure

**DOI:** 10.1007/s10695-025-01468-0

**Published:** 2025-02-26

**Authors:** Cailin van Staden, Karin Finger-Baier, David Weinshenker, Tarryn L. Botha, Linda Brand, De Wet Wolmarans

**Affiliations:** 1 Center of Excellence for Pharmaceutical Sciences, Department of Pharmacology, North-West University, 11 Hoffman Street, Potchefstroom, 2520 South Africa; 2 Department Genes - Circuits - Behavior, Max Planck Institute for Biological Intelligence, Martinsried, Germany; 3 Department of Human Genetics, Emory University School of Medicine, 615 Michael St., Whitehead 301, Atlanta, GA 30322 USA; 4 Department of Zoology, University of Johannesburg, Auckland Park, Johannesburg, 2006 South Africa

**Keywords:** Conspecific alarm substance, Zebrafish, Stress, Anxiety, Risk-taking

## Abstract

Zebrafish (*Danio rerio*) represents a complementary pre-clinical model in stress and anxiety research. Conspecific alarm substance (CAS), an alarm pheromone secreted by injured fish, acts as a warning signal and modulates fear responses. Given their schooling nature and that injury precedes CAS release, varying fresh CAS concentrations extracted from different numbers of CAS-donating zebrafish may uniquely influence trauma-related behaviours. Thus, we investigated the behaviour of juvenile and adult zebrafish exposed to traumatic stress protocols, in the presence of CAS extracted from varying numbers of donating zebrafish. Juveniles were assessed for anxiety and boldness in the light–dark and open field tests (LDT and OFT), while adults were assessed in the novel tank test (NTT) and novel OFT (nOFT). We found that (1) trauma minimally impacted juvenile behaviour regardless of donor-derived CAS concentrations, (2) trauma-exposed adults displayed reduced exploration and heightened risk-taking behaviours in the NTT and nOFT compared to control-exposed fish, (3) NTT and nOFT freezing behaviours were distinctly emulated in adult fish and (4) post-trauma behaviour in adults was influenced by the number of donors. Therefore, CAS concentration as determined by donor number has age-related effects on anxiety- and risk-taking behaviours in trauma-exposed zebrafish, a valuable finding for studies utilising fresh CAS as a stress trigger. While we did not directly investigate CAS concentration through serial dilution, our data are of significant translational and ethological relevance, highlighting the importance of in-house method standardization in stress-related studies utilizing fresh CAS as an alarm cue.

**Supplementary Information:**

The online version contains supplementary material available at 10.1007/s10695-025-01468-0.

## Introduction

Stress, a state of threatened homeostasis (Agorastos et al. 2019; Chrousos 2009; Chrousos & Gold 1992; Pervanidou & Chrousos 2018), is regarded as a normal adaptive physiological response that is essential for an organism’s survival (Chrousos 2009; Juruena et al. 2021; Pechtel & Pizzagalli 2011; Schiavone et al. 2015; Silberman et al. 2016). However, when stress becomes excessive, it can surpass the organism’s natural adaptive capacities, triggering maladaptive processes (Agorastos et al. 2019; Chrousos 2009; Pervanidou & Chrousos 2018; Silberman et al. 2016). In humans, this can arise from trauma exposure, resulting in an increased vulnerability to developing neuropsychiatric disorders, especially anxiety disorders, depression and post-traumatic stress disorder (PTSD) (Agorastos et al. 2019; Belujon & Grace 2015; Chrousos 2009; DeRosse & Barber 2020; Musazzi et al. 2017; Schiavone et al. 2015).

Considering the unpredictable nature and occurrence of severely stressful, i.e., traumatic, events, the study of human stress often poses ethical and methodological constraints (Bale et al. 2019; Nestler & Hyman 2010). Thus, our understanding gains much from research in preclinical model systems, most of which are developed in rodent strains (Atrooz et al. 2021; de Abreu et al. 2021; Demin et al. 2021; Molet et al. 2014; Verbitsky et al. 2020). Complementing existing murine models and clinical studies, zebrafish (*Danio rerio*) has emerged as a prominent preclinical model organism that is increasingly utilised in translational studies of stress and anxiety (Collier et al. 2017; de Abreu et al. 2021; Griffiths et al. 2012; Stewart et al. 2014; Van Staden et al. 2025). For example, zebrafish, both in the juvenile and adult stages, share extensive genetic, anatomical and physiological homology with mammals, including rodents and humans (Howe et al. 2013; Kalueff et al. 2014a; Kalueff et al. 2014b; Maximino et al. 2015; Stewart et al. 2014). Specifically, zebrafish possess a neuroendocrine stress axis, the hypothalamic-pituitary-interrenal (HPI) axis, which exhibits significant organisational and functional similarity to the mammalian hypothalamic–pituitary–adrenal (HPA) axis (Alsop & Vijayan 2009; Demin et al. 2021; Steenbergen et al. 2011). Additionally, in both humans and zebrafish, cortisol and noradrenaline are key facilitators of the stress response (Demin et al. 2021; Madaro et al. 2020; Schreck & Tort 2016; Steenbergen et al. 2011). Their stress-related behavioural repertoire is also broadly conserved and well-characterised (Collier et al. 2017; de Abreu et al. 2021; Kalueff et al. 2013; Kysil et al. 2017; Sabadin et al. 2022). Specifically, juvenile and adult zebrafish offer a range of quantifiable anxiety-like behaviours, such as bright light avoidance, startled freezing, bottom dwelling and thigmotaxis (Collier et al. 2017). Conversely, zebrafish lend themselves well to the study of bidirectional behavioural separation, since subpopulations of fish can be resistant to anxiogenic stimuli and show increased risk-taking behaviour (Ariyomo et al. 2013; Dahlbom et al. 2011; Wisenden et al. 2011; Wright et al. 2003), a behavioural trait that commonly parallels decreased anxiety (Giorgetta et al. 2012; Smith et al. 2016).

Notably, fish also convey danger signals to conspecifics via alarm pheromones such as conspecific alarm substance (CAS), which is released by injured fish (Chivers et al. 2012; Døving & Lastein 2009; von Frisch 1938). CAS is synthesised in the epidermal cells of zebrafish and is released when cell membranes are damaged (Chivers et al. 2012; Maximino et al. 2019; Oliveira et al. 2014). This results in a stressogenic signal to nearby conspecifics, which detect CAS through olfaction (Egan et al. 2009; Lucon-Xiccato et al. 2020; Maximino et al. 2018; Quadros et al. 2016; Speedie & Gerlai 2008). That zebrafish release CAS upon injury is important, since the premise is that, ethologically viewed, CAS acts as a warning signal only during traumatic experiences. Also, from an evolutionary and ethological perspective, it is unlikely that alarm signals in fish carry the same psychobiological valence as do trauma-related sensory stimuli in mammals; rather, it is proposed that its role as an alarm signal coincidentally evolved secondary to its function as a self-preservatory immunogenic modulator (Chivers et al. 2007). Notably, zebrafish as young as 5 to 7 days post-fertilisation (dpf) are capable of sensing and responding to CAS originating from adult individuals (Jesuthasan et al. 2021). Since CAS is detected peripherally, neurobiological CAS-triggered contextualisation of traumatic events is promulgated via bottom-up (i.e., peripheral-to-central) mechanisms, as opposed to the mainly top-down psychobiological stress signalling in mammals (Compas 2006; Theron et al. 2023). In the laboratory, CAS is commonly employed as a biochemical stressor in translational studies of anxiety, either in isolation or in combination with other stressors (Lucon-Xiccato et al. 2020; Song et al. 2018; Speedie & Gerlai 2008; Theron et al. 2023; Van Staden et al. 2025). When administered alone, CAS exposure induces anti-predatory and anxiety-like responses (Egan et al. 2009; Speedie & Gerlai 2008) as well as heightened arousal 24 h after exposure (Lima et al. 2015).

CAS has been the subject of scientific inquiry for nearly a century (von Frisch 1938). However, it is only recently that the chemical composition of CAS has been elucidated. Two recent studies have shown that the parallel detection of two molecules, ostariopterin and daniosulfate (Masuda et al. 2024) (also known as 5α-cyprinol sulfate and 5α-daniol sulfate; Li et al. 2023a, b), is responsible for the typical alarm reactions in zebrafish. However, from an ethological perspective, it remains unclear to what extent the degree of injury, or alternatively, the number of injured fish in a school contributes to trauma-related behaviour in this species. Indeed, most studies to date have relied on freshly extracted CAS using various methods and different numbers of CAS-donating zebrafish, likely contributing to inconsistent data relating to CAS-induced anxiety-like responses (Egan et al. 2009; Lima et al. 2016; Maximino et al. 2018; Nathan et al. 2015; Quadros et al. 2016). Importantly, methodological variation is significant, with some studies using as few as one donor (Maximino et al. 2018), while others use eight (Theron et al. 2023) or 10 (Speedie & Gerlai 2008).

Therefore, the goal of this research was to investigate the influence of different numbers of CAS-donating zebrafish, on the trauma-related anxiety- and risk-related behavioural responses of juvenile and adult zebrafish exposed to CAS, in combination with age-appropriate traumatic stress protocols. Our objective was not to isolate the effects of either stress or CAS, as this has been extensively studied before (de Abreu et al. 2021; Demin et al. 2021; Egan et al. 2009; Quadros et al. 2016), but rather to understand how different numbers of CAS donors would affect the behavioural outcomes of trauma-exposed fish.

## Materials and methods

### Fish

A total of 240 randomly chosen wild-type long-fin zebrafish of both sexes (120 juvenile, 30 ± 3 dpf, ± 10 mm standard length (SL) and 120 adult, 3–5 months old, ± 30–40 mm SL) were used as test subjects in this investigation. Another 50 long-fin adult zebrafish (4 months old, 40 mm SL, both sexes) were euthanised and used as CAS donors (refer to paragraph 2.4). Additionally, 24 tiger barbs (*Puntigrus tetrazona*) were used as live aggressors to test adult zebrafish for risk-taking behaviour (refer to paragraph 2.5). The progenitor stock for zebrafish and the experimental tiger barb stock were obtained from Wave Aquatics (Pretoria, South Africa) and housed at the National Aquatic Bioassay Facility (NABF) of the North-West University (NWU), Potchefstroom, South Africa.

Larval zebrafish were bred in-house from multiple breeding pairs of fish, by means of an iSpawn® (Zebtech®, Tecniplast®) tank. Eggs were collected and kept in Petri dishes (9 cm × 2 cm) filled with E3 media, with approximately 50–60 larvae per Petri dish. These dishes were placed in an incubator equipped with a lighting timer and maintained at 28.5 °C until the larvae reached 5–7 dpf, ensuring swim bladder inflation, which typically occurs around 4 dpf. Every day, Petri dishes were monitored for deformed and dead fish, which were removed. Fifty per cent of the E3 media was replaced daily. Between 5 and 7 dpf, fish were transferred to 8-L acrylic tanks (Zebtech®, Tecniplast®) equipped with 300-µm mesh back wall inserts to prevent larvae from exiting the tank (± 50 larvae per tank; excess larvae were bred to account for unexpected deaths). Larvae were raised until 30 dpf ± 3 days at a temperature of 28 ± 1 °C and a pH of 7.4, with continuous aeration applied and daily water changes (75% of the total volume) performed until experimentation (Aleström et al. 2020). Fish were fed four times daily from 5 through 13 dpf, alternating between freshwater rotifers and fry food (ZM-000, ZM Ltd.). From 14 through 29 dpf, live *Artemia salina* (brine shrimp nauplii; Ocean Nutrition™, South Africa) and fry food (ZM-000, ZM Ltd.) were given alternately. Experimental adults were merely selected from the housing stock of the NABF and kept in groups of 24 fish per 8-L tank until experimentation (ZebTec® zebrafish housing system, Techniplast®, Varese, Italy; pH: 7; conductivity: 600 μS; 26 ± 0.1 °C; 7.2 mg O_2_/L). Adults were fed fry food (ZM-400, ZM Ltd.) three times daily by means of an automated Tritone® feeding system. All tiger barbs were housed in a separate aerated and temperature-maintained tank (80 L; 24 ± 0.1 ˚C; pH: 7) and fed once daily with either bloodworms (Ocean Nutrition™, South Africa) or Tetramin™ flakes. All fish, irrespective of age, were maintained on a normal 12-h light/dark cycle (06h00/18h00). All procedures and experimental methods were approved by the AnimCare Research Ethics Committee of North-West University (approval number: NWU-00754–22-A5; Committee Registration Nr. AREC-130913–015; Approved: 01 June 2022).

### Experimental design

One day before traumatic stress (or control) exposure (experimental day 0), experimental zebrafish were randomly allocated to five juvenile and adult groups (*n* = 24 per group). Juvenile zebrafish were transferred into 3-L holding tanks, whereas adult zebrafish were transferred into new 8-L tanks.

**Fig. 1:**
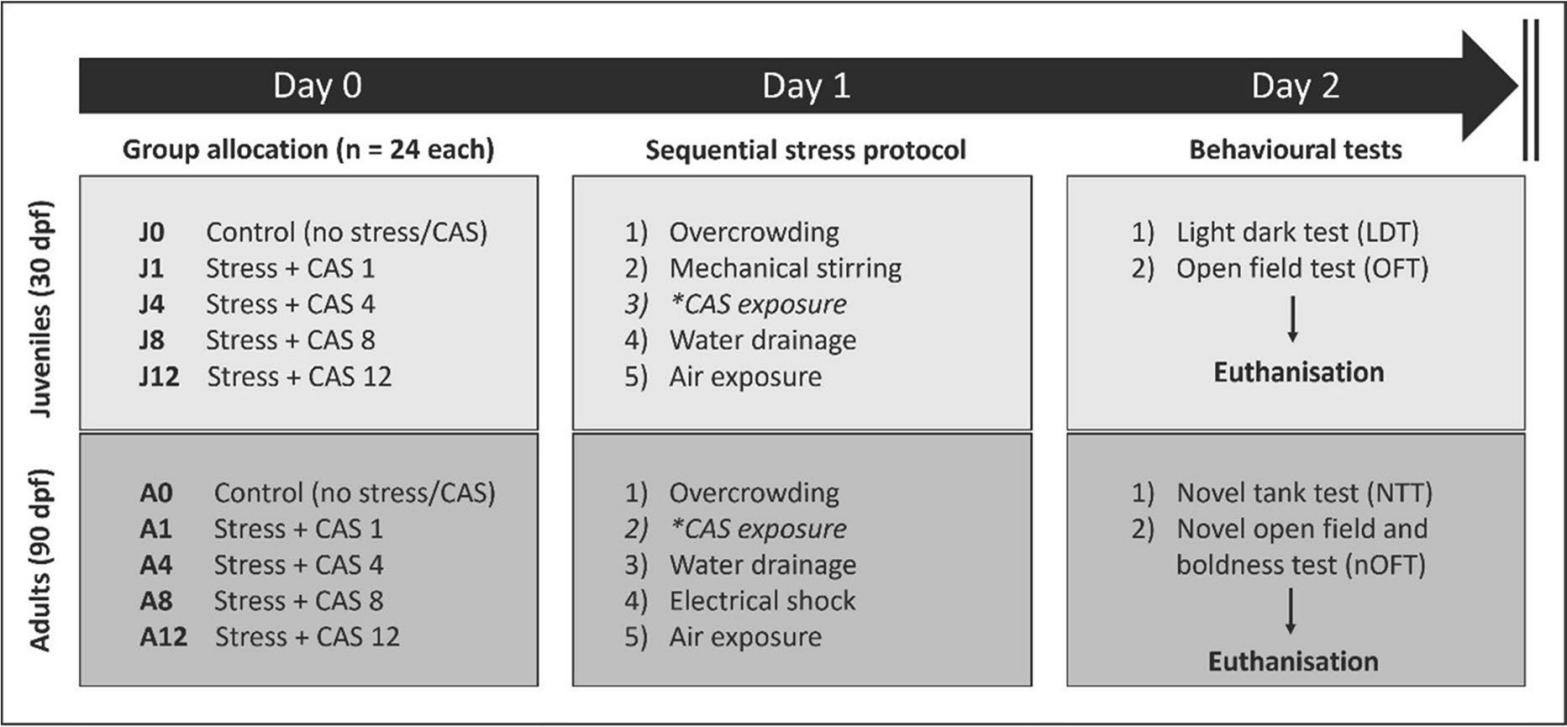
Experimental layout. *As per group-specific donor fish number. dpf: day post-fertilisation; CAS: conspecific alarm substance

One cohort in each age group (group J0—juvenile or group A0—adult; Ctrl) was used as behavioural controls (no stress exposure) (Fig. 1). The other four groups were all subjected to the same juvenile and adult stressors, respectively. However, each group was stressed in the presence of a different CAS concentration that increased between groups, i.e., groups J1 and A1 (CAS concentrate extracted from one fish; CAS 1), groups J4 and A4 (CAS concentrate extracted from four fish; CAS 4), groups J8 and A8 (CAS concentrate extracted from eight fish; CAS 8) and groups J12 and A12 (CAS concentrate extracted from 12 fish; CAS 12) (refer to paragraph 2.4). All CAS concentrates were freshly prepared immediately before use in a volume of 10-mL ultrapure water (Milli-Q®), which was added to 2 L of system water during execution of the stress protocols.

Zebrafish in each respective group were group-exposed to either a no-stress (groups J0 and A0) or traumatic stress (groups J/A 1, 4, 8 and 12) protocol on experimental day 1. After stress exposure, zebrafish were returned to their respective home tanks until behavioural testing the following day (experimental day 2). On day 2, individual zebrafish from each respective group were gently transferred to the first of two behavioural tests. Juvenile zebrafish were subjected to light–dark (light–dark test; LDT) and adult zebrafish to novel tank testing (novel tank test; NTT) (Fig. 1) first. Following completion of these tests, juvenile fish were immediately subjected to open field testing (open field test; OFT), while adult fish were assessed in a novel version of the open field that was designed to measure both open field avoidance and aggressor-directed risk-taking behaviour (novel open field and boldness test; nOFT). Free exploration of each behavioural test was allowed for a 6-min videotaped session. Behavioural testing sessions commenced at 08:00 each day. To avoid satiety-related influences on testing performance, all zebrafish were fed 1 h before the first behavioural session began. After the last test was completed, zebrafish were gently netted from the respective arenas and euthanised via immersion in liquid nitrogen (Simonetti et al. 2016).

### Traumatic stress protocol for juvenile and adult zebrafish

#### Apparatus

**Fig. 2:**
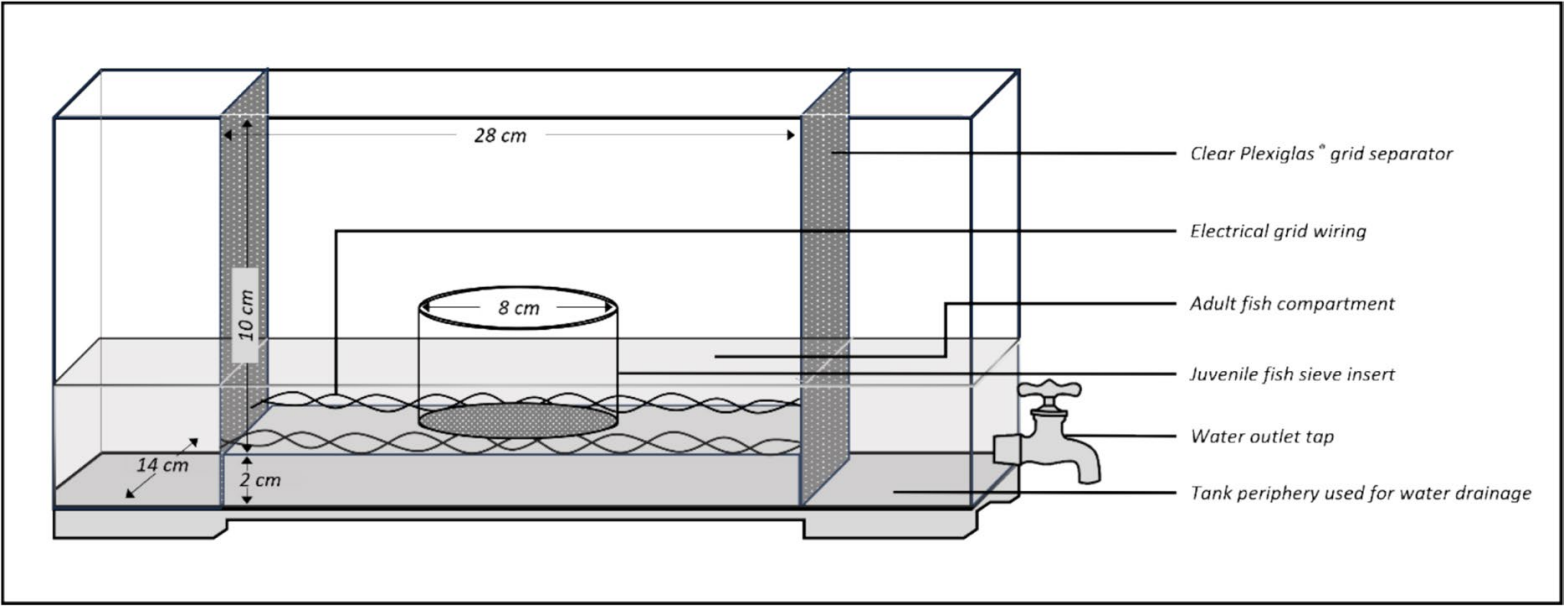
Stress exposure apparatus used for juvenile and adult zebrafish

The main stress exposure apparatus used for both juvenile and adult zebrafish consisted of a rectangular tank constructed from clear Plexiglas® (28 cm × 14 cm × 10 cm) (Fig. 2). The floor of the tank comprised two levels, i.e. an internal (false) floor and a base floor (solid floor). The internal floor of the apparatus consisted of a Plexiglas® grid to facilitate water drainage while the solid floor (2 cm below the internal floor) attached and sealed the tank to its walls (Theron et al. 2023; Van Staden et al. 2025). The space between the false inner floor and the solid floor was used as a conduit for water drainage. A grid of electrical wiring that was able to conduct electrical current (peak voltage: 1300 V; peak current: 240 mA; discharge time: 100 ms; tank resistance: 5 kΩ) uniformly throughout the entire tank was affixed to the terminal walls of the tank (Van Staden et al. 2025). The exposure tank was used as is for the adult stress protocol. However, for juvenile fish, a transparent circular sieve (8 cm diameter) was inserted into the exposure tank (Fig. 2) to reduce the exposure volume and ensure overcrowding. After each group of fish was stressed, the tank and, where applicable the insert, was washed with a salt solution to eliminate any residual odours and traces of CAS from previous sessions.

#### General background

We have previously developed an ethologically relevant and life-threatening traumatic stress protocol for application in trauma-related translational studies in zebrafish (Theron et al. 2023). Although said protocol was validated in adult fish only, the present work aimed to extend this approach to juvenile zebrafish, to enable future research into the impact of early-life trauma in this species. This approach included a combination of different stressors that have previously been applied in zebrafish aged 5 to 21 dpf, including overcrowding (Fontana et al. 2021a, b; Fontana et al. 2021a, b), mechanical stirring (Fontana et al. 2021a, b; Fontana et al. 2021a, b; Wu et al. 2023), water drainage (Fontana et al. 2021a, b; Fontana et al. 2021a, b; Lopez-Luna et al. 2017) and air exposure (Hare et al. 2021; Lopez-Luna et al. 2017). Further, CAS was used as an additional stressor (Lopez-Luna et al. 2017) as explained earlier. However, considering the lack of knowledge regarding the influence of electrical shock exposure in juvenile fish, we excluded this stressor from juvenile fish experiments for ethical reasons. For adult fish, the stressor was applied as previously described (Piato et al. 2011; Ramsay et al. 2006; Theron et al. 2023), with slight modification to allow for group exposure.

#### Juvenile stress protocol

To elicit a traumatic stress response in juvenile zebrafish, we combined a series of age-appropriate stressors, previously employed in juvenile zebrafish studies, to create the juvenile stress protocol. On experimental day 1, complete groups of 24 juvenile zebrafish were transferred from their holding tanks to clear containers (18 cm × 11 cm × 8 cm) filled with 400 mL of system water, where they underwent overcrowding for 50 min (Fontana et al. 2021a, b; Fontana et al. 2021a, b). Subsequently, fish were gently transferred to the circular sieve which enclosed approximately 125-mL system water in the exposure tank. This transition maintained and intensified the overcrowded conditions within the sieve, now placed inside the exposure tank, for the entire duration of the stress protocol. The exposure tank contained 2 L of system water, 10 mL of the appropriate CAS concentration (see below) and the euthanised CAS-releasing fish (Lopez-Luna et al. 2017; Theron et al. 2023). The inclusion of the euthanized donor(s) served to simulate a visual life-threatening context similar to a natural predation scenario, thereby strengthening the ethological relevance of the protocol and contextualising CAS exposure as an alarm signal released during actual traumatic events (Theron et al. 2023). After 10 min in this scenario, fish were subjected to 2 min of mechanical stirring using a Pasteur pipette (Fontana et al. 2021a, b; Fontana et al. 2021a, b; Wu et al. 2023), after which the water level was slowly drained over 3 min until all fish were air-exposed (Fontana et al. 2021a, b; Fontana et al. 2021a, b; Lopez-Luna et al. 2017). Zebrafish were then entirely air-exposed for 1 min (Hare et al. 2021; Lopez-Luna et al. 2017) and subsequently transferred back to their home tanks. Total ammonia nitrogen (TAN) levels were kept normal as a function of between-exposure replacement of exposure water with fresh system water as described previously (Theron et al. 2023).

#### Adult stress protocol

To elicit a traumatic stress response in adult zebrafish, we combined a series of age-appropriate stressors, previously employed in adult zebrafish studies. Adult zebrafish were transferred in complete groups of 24 fish to the exposure tank containing 2 L of system water (without the sieve insert). They remained in this overcrowded condition for 50 min (Piato et al. 2011) prior to the introduction of subsequent stressors. Importantly, the overcrowded condition persisted throughout the entire duration of the stress protocol. After the initial 50 min, 10 mL of the appropriate CAS-containing extracts was introduced to the tank, together with the euthanised CAS-releasing fish (Theron et al. 2023). Again, zebrafish were left for 10 min in this scenario, after which the water was drained over 3 min (Piato et al. 2011), and a single electrical shock was applied 90 s after the onset of water drainage (Theron et al. 2023). Zebrafish were then air exposed for 2 min before they were transferred back to their home tanks (Ramsay et al. 2009). Exposure water was again replaced between each exposure session.

### CAS extraction

Since no standardised method of CAS extraction has been described, procedures previously described by Egan et al. (2009), Maximino et al. (2018), Quadros et al. (2016), Speedie and Gerlai (2008) and Theron et al. (2023) were used as a guideline. Fresh CAS was extracted from the appropriate number of CAS-donating zebrafish (either 1, 4, 8 or 12 fish), immediately prior to application of the respective stress protocols. CAS-donating zebrafish were euthanised by means of a rapid blow to the head followed by decapitation with a scalpel on a solid, non-porous surface. Fish were gently dried with paper towel to remove excess system water and blood and placed in a clean Petri dish. To damage the epidermal cells, 10 shallow incisions (approximately 0.5 cm long) were made on each side of the zebrafish, starting from behind the gills and extending to just before the caudal fins, using a standard razor blade. Ten-millilitre ultrapure water was then added to the Petri dish and mixed to fully cover the lacerated portions of the donor fish. Euthanised fish were left in this condition for 10 min. Thus, the concentration of CAS was ‘standardised’ as the amount of CAS released by the specified number of zebrafish into 10-mL ultrapure water over 10 min. The euthanised donors, together with the 10-mL CAS-containing liquid, were then added to the exposure tank as described earlier, taking into consideration that such addition was made prior to inserting the sieve, in the case of juvenile stress exposure.

### Behavioural tests

#### General background

All behavioural testing arenas were either placed on (juvenile LDT and OFT or adult nOFT) or in front of (adult NTT) an infrared backlight to facilitate automated behavioural tracking (see below). A digital video camera was mounted either above or in front of the respective test apparatuses. All recordings were analysed by means of the EthoVision® XT 14 digital tracking software (Noldus® Information Technologies, Wageningen, The Netherlands). The initial sample size for juvenile and adult zebrafish was *n* = 120, respectively; however, the final sample size for juvenile fish was *n* = 119 (one fish was excluded from group J12 due to accidental injury that occurred during handling) and *n* = 117 for adult fish (three fish were excluded; two from group A1 and one from group A4 due to fish jumping from the testing arenas).

#### Juvenile LDT

The LDT has been extensively used to assess general anxiety, as well as to screen for anxiogenic and anxiolytic drugs (Collier et al. 2017; Kysil et al. 2017; Maximino et al. 2010). The LDT was performed in standard transparent plastic Petri dishes (8.5 cm in diameter). Two thirds of the diameter of the Petri dish consisted of a clear background. The other third was black. To obscure external cues, the side perimeters of the Petri dishes were rendered opaque with white and black tape which covered the sides along the corresponding floor colours of the dish. Apart from the larger area of the Petri dish, a virtual border zone (1 cm from the sides) was delineated to score thigmotaxis. Before each assessment, each Petri dish was filled with 45 mL of system water. During any given assessment session, 12 zebrafish were individually transferred to 12 Petri dishes (12 zebrafish tested simultaneously) whereby free exploration was allowed for a 6-min, videotaped session. Increased time spent in the black zone and in the entire border zone (expressed as the percentage of the total testing time) was regarded as anxiety-like behaviour.

#### Juvenile OFT

Slightly deviating from the juvenile LDT, the OFT is commonly used to assess general locomotor activity and novelty-evoked anxiety-like behaviours (Collier et al. 2017; Maximino et al. 2010). The OFT apparatus and test setup was the same as that used for the LDT (8.5 cm in diameter). A similar virtual border zone (1 cm from the sides) was delineated to score thigmotaxis. However, in this instance, the entire floor was clear, while the entire side was covered with white opaque tape. Total distance travelled was used to assess changes in locomotion. Increased time spent along the border of the Petri dish was regarded to be indicative of anxiety-like behaviour.

#### Adult NTT

The NTT is used to measure locomotion, anxiety-like behaviour and boldness in zebrafish (Collier et al. 2017). On any given day of testing, zebrafish were individually placed in a novel tank (25 cm × 10 cm × 7 cm) constructed from opaque white Plexiglas®, except for the front- and back-facing panels, which was left clear to facilitate automated scoring. The tank was filled with the 1300-mL system water and virtually divided into two zones, i.e. top and bottom, to provide a detailed assessment of vertical swimming activity. Locomotor activity was measured as a general indicator of activity. More time spent in and entries made into the top zone of the tank were indicative of a lesser degree of anxiety and increased risk-taking behaviour (Collier et al. 2017).

#### Adult nOFT

**Fig. 3:**
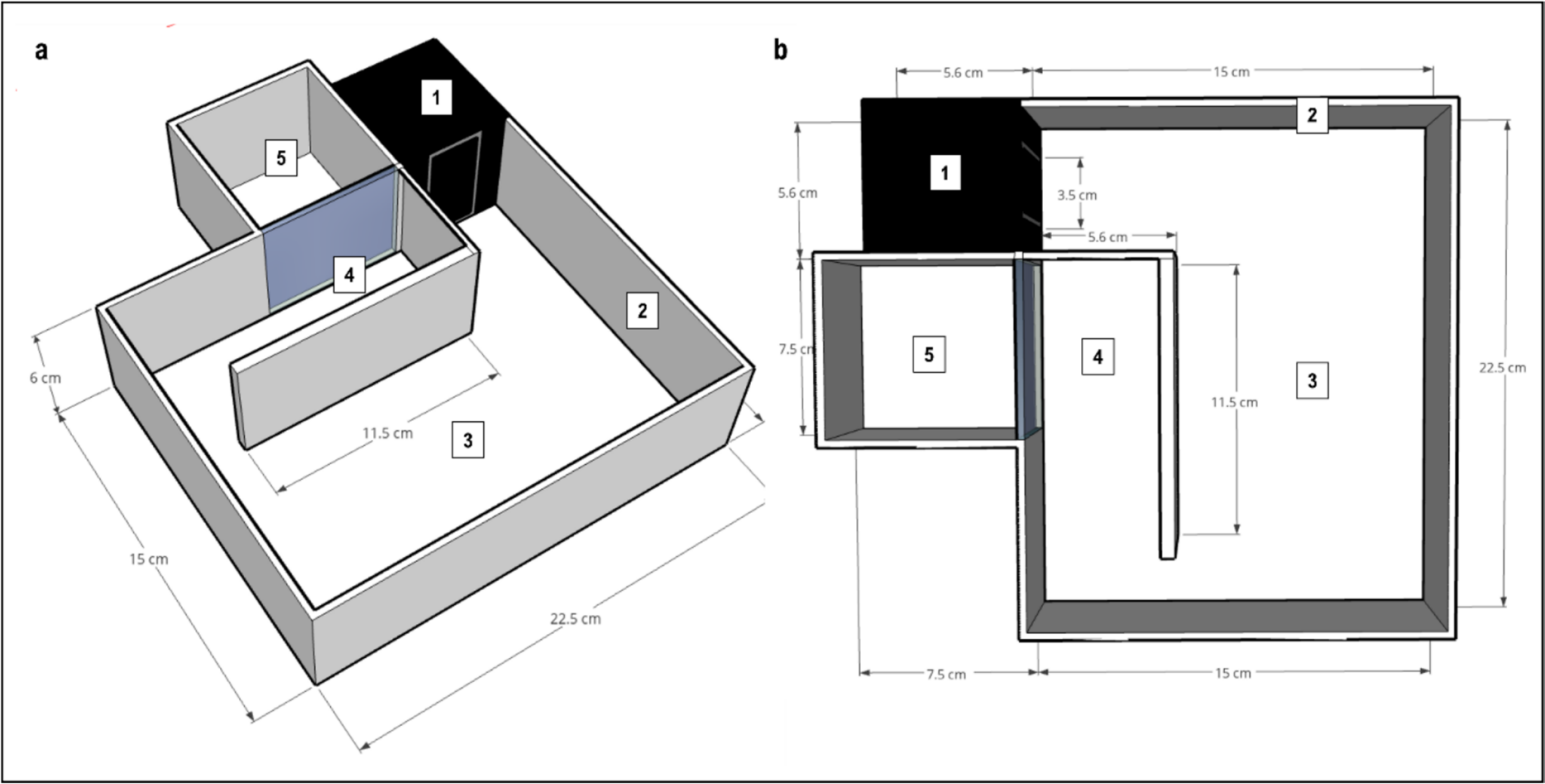
Schematic representation of the novel open field and boldness test (nOFT); **a** side view and **b** top view. (1) dark compartment to measure scototaxis, (2) maze walls to measure thigmotaxis, (3) large open area to measure anxiolytic responses, (4) a proximity area and (5) an aggressor compartment

A novel version of a standard open field, the nOFT maze, was designed for the present purpose and used to simultaneously assess light–dark preference, open field ambulation and aggressor-directed, i.e. risk-taking, behavioural response (Van Staden et al., 2025). The maze was constructed from clear (floor) and opaque white (walls) Plexiglas® (22.5 cm (l) × 15 cm (w) × 6 cm (h); Fig. 3) and physically divided into four compartments, viz*.* (i) a dark compartment (starting box), constructed from black, infrared-translucent Plexiglas® (5.6 cm × 5.6 cm × 6 cm); (ii) a large open area (22.5 cm × 15 cm × 6 cm); (iii) a passageway (11.5 cm × 5.6 cm × 6 cm) and (iv) an aggressor-containing compartment (7.5 cm × 7.5 cm × 6 cm), the latter which was only visible and accessible via the passageway. The dark compartment had a removable sliding door, which closed off the opening into the larger maze at the onset of experimentation. The aggressor-containing compartment, always containing two tiger barbs, was also constructed from opaque white Plexiglas® with a clear floor. The side of this compartment adjoining the larger maze was constructed from a clear Plexiglas® grid, enabling experimental zebrafish to see into and visually interact with the aggressors when facing this wall; however, fish could not interact physically. Zebrafish had to enter the proximity area directly in front of the aggressor compartment; before seeing or interacting with aggressors was possible. A virtual border zone was drawn around the open field area of the maze to measure thigmotaxis. At all times, the maze was filled to a depth of 5 cm with system water. Zebrafish were individually placed into the starting box of each maze and allowed to freely explore the maze for 6 min. On each day of testing, maze water was changed after every two trials (van der Westhuizen et al. 2023; Van Staden et al., 2025). Additionally, aggressor fish were rotated with new individuals after every two trials to minimise stress. Locomotor activity was measured as a general indicator of activity. Increased time spent in the dark compartment or within a virtual border zone delineated 1 cm from the sides of the open field (thigmotaxis) was regarded to represent anxiety-like behaviour. Conversely, increased time spent in the centre of the open field was indicative of decreased anxiety-like behaviour, while aggressor-interaction, reflected by increased time spent in the proximity area, was applied as a measure of boldness/risk-taking.

### Statistical analysis

Statistical analyses were performed with the GraphPad Prism® 8.0.1 software. Considering the degree of variance displayed in the behaviour of zebrafish in each group, Kruskal–Wallis tests were applied to determine if CAS concentration impacted the behavioural output of juvenile and adult zebrafish, as measured against the control. Post hoc pairwise comparisons were performed using Dunn’s procedure. Statistical significance was set at *p* < 0.05 for all analyses. Where applicable, pairwise comparisons were informed with calculations of Cohen’s *d* effect size to establish the magnitude of the effects observed. Effect sizes were considered large at 0.8 or greater (Cohen 1988). All graphs and figures were prepared with GraphPad Prism® version 10 (GraphPad®, San Diego, USA).

## Results

### Juvenile behaviour

#### LDT (Fig. 4; Supplementary Table 1)

**Fig. 4:**
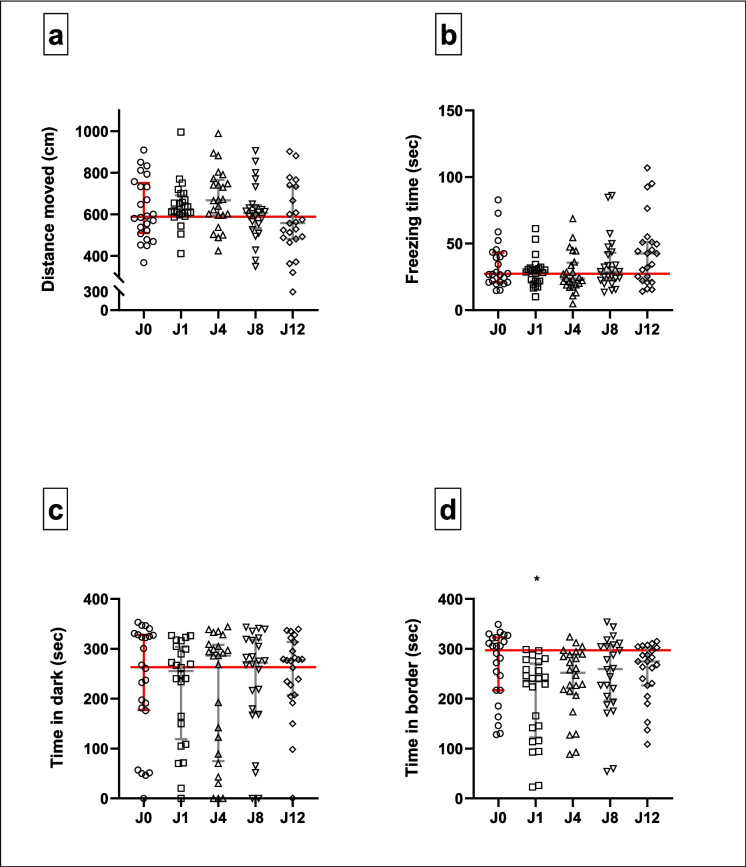
Behavioural responses of juvenile zebrafish in the LDT under different conditions: control conditions (J0) and traumatic stress exposure paired with varying CAS concentrations (J1–12). Horizontal lines represent the median values of control-exposed fish. Kruskal–Wallis test followed by Dunn’s procedure for multiple comparisons (*n* = 24 per group, except group J12 where *n* = 23). Data are presented as median with interquartile range. *p < 0.05

In terms of total distance swam in the LDT (Fig. 4a), median scores did not differ significantly between the different concentrations of CAS exposure (*H*[4] = 7.3, *p* = 0.12). There was also no difference between the median times spent freezing (*H*[4] = 6.6. *p* = 0.16, Fig. 4b) or time spent in the dark third of the Petri dish (*H*[4] = 1.4. *p* = 0.84, Fig. 4c). However, the median times spent in the entire border zone of the Petri dish differed significantly between groups (*H*[4] = 11.8, *p* = 0.02), with juvenile zebrafish exposed to CAS extracted from a single fish, spending significantly less time in the border (235 s), compared to non-stressed fish (298 s; *p* = 0.01, *d* = 0.8, 95CI [− 1.432, − 0.250], Fig. 4d). Descriptive statistics are provided in supplementary Tables 1A—D.

#### OFT (Fig. 5; Supplementary Table 2)

**Fig. 5:**
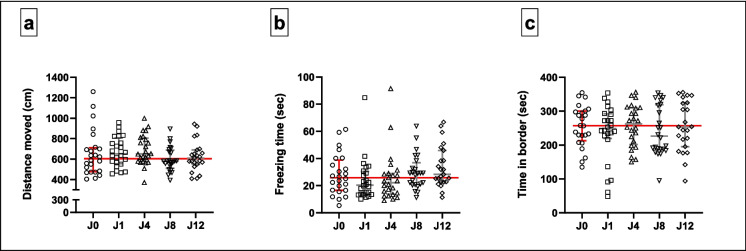
Behavioural responses of juvenile zebrafish in the OFT under different conditions: control conditions (J0) and traumatic stress exposure paired with varying CAS concentrations (J1–12). Horizontal lines represent the median values of control-exposed fish. Kruskal–Wallis test followed by Dunn’s procedure for multiple comparisons (*n* = 24 per group, except group J12 where *n* = 23). Data are presented as median with interquartile range

CAS concentration affected neither the total distance moved (*H*[4] = 5.5, *p* = 0.24, Fig. 5a), nor the total time spent in the border zone of the OFT Petri dish (*H*[4] = 1.2, *p* = 0.88, Fig. 5c). Further, while stress-paired CAS concentration impacted the time spent freezing (*H*[4] = 9.5, *p* = 0.05, Fig. 5b), no statistically significant differences were demonstrated between any of the exposure groups. Descriptive statistics are provided in supplementary Tables 2A—C.

### Adult behaviour

#### NTT (Fig. 6; Supplementary Table 3)

**Fig. 6:**
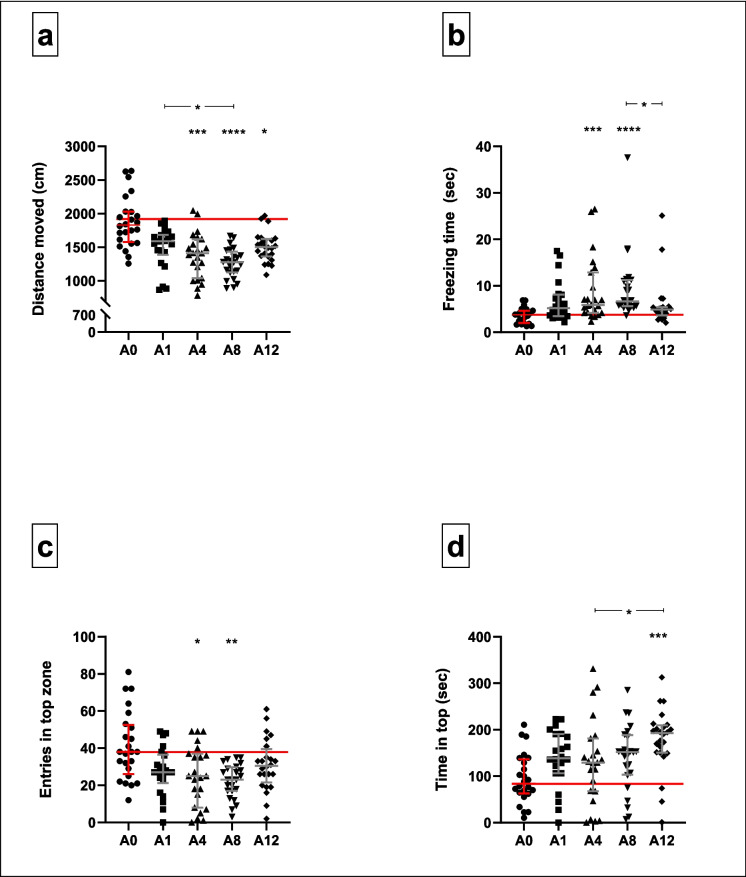
Behavioural responses of adult zebrafish in the NTT under different conditions: control conditions (A0) and traumatic stress exposure paired with varying CAS concentrations (A1–12). Horizontal lines denote the median values of control-exposed fish. The Kruskal–Wallis test followed by Dunn’s procedure for multiple comparisons (*n* = 24 per group, except group A1 where *n* = 22 and A4 where *n* = 23). Data are presented as median with interquartile range. *p < 0.05; **p < 0.01; ***p < 0.001; ****p < 0.0001

Trauma-paired CAS exposure broadly impacted all NTT outcomes (Fig. 6). Median scores differed significantly between groups with respect to the total distance swam (*H*[4] = 36.9, *p* < 0.0001, Fig. 6a), time spent freezing (*H*[4] = 31.7, *p* < 0.0001, Fig. 6b), number of entries into the top zone (*H*[4] = 16.3, *p* = 0.003, Fig. 6c) and the time spent in the top zone of the tank (*H*[4] = 19.7, *p* = 0.0006, Fig. 6d).

With respect to the total distance swam, the median scores of all stress-exposed groups, except the A1 group, were significantly lower compared to that of control-exposed fish (1828 cm) (A1: 1587 cm, *p* = 0.06, *d* = 1.1, 95CI [− 1.717, − 0.473]; A4: 1411 cm; *p* = 0.0001, *d* = 1.4, 95CI [− 2.033, − 0.752]; A8: 1278 cm, *p* < 0.0001, *d* = 2.0, 95CI [− 2.658, − 1.270] and A12: 1502 cm, *p* = 0.01, *d* = 1.2, 95CI [− 1.814, − 0.581]). Fish in groups A4 (5.9 s, *p* = 0.0007, *d* = 1.1, 95CI [0.433, 1.656]) and A8 (6.7 s, *p* < 0.0001, *d* = 1.1, 95CI [0.514, 1.736]) also spent significantly more time freezing, compared to control-exposed fish. The median number of top zone entries made by fish in groups A4 (25 entries, *p* = 0.03, *d* = 0.9, 95CI [− 1.528, − 0.322]) and A8 (23 entries, *p* = 0.002, *d* = 1.3, 95CI [− 1.890, − 0.645]) was significantly less compared to those made by control exposed fish (38 entries), while fish in group A12 spent significantly more time in the top zone of the arena (193 s), compared to non-stress-exposed fish (84 s, *p* = 0.0002, *d* = 1.4, 95CI [0.733, 1.994]). Although narrowly missing statistical significance, fish in group A8 trended towards the same (152 s, *p* = 0.17, *d* = 0.7, 95CI [0.144, 1.315]). Descriptive statistics are provided in supplementary Tables 3A—D.

#### nOFT (Fig. 7; Supplementary Table 4)

**Fig. 7:**
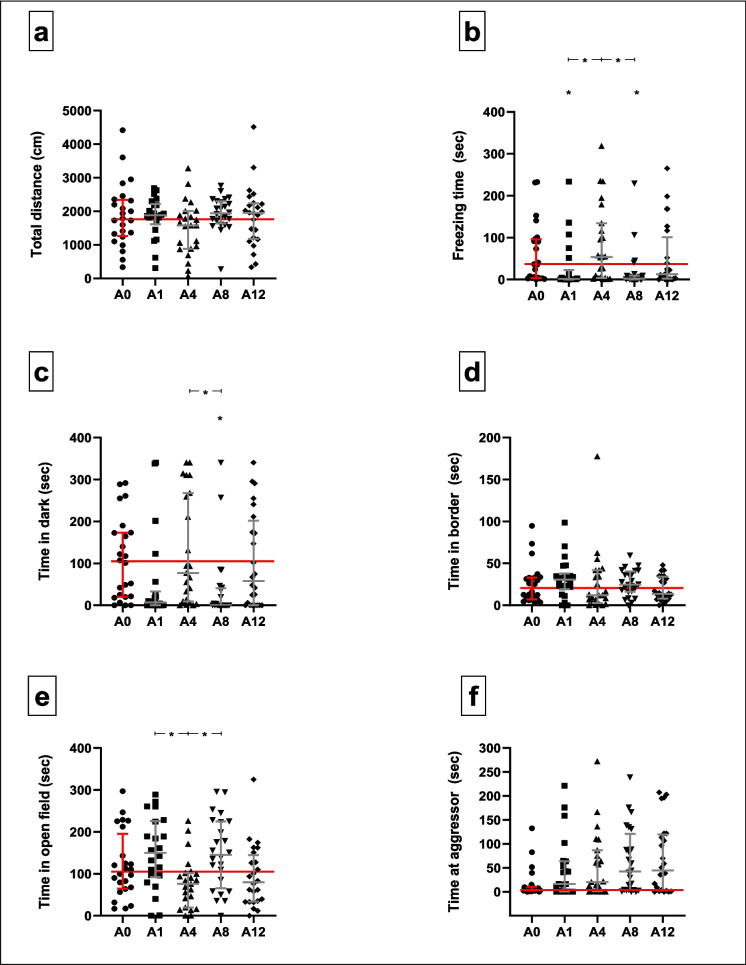
Behavioural responses of adult zebrafish in the nOFT under different conditions: control conditions (A0) and traumatic stress exposure paired with varying CAS concentrations (A1–12). Horizontal lines denote the median values of control-exposed fish. Kruskal–Wallis test followed by Dunn’s procedure for multiple comparisons (*n* = 24 per group, except group A1 where *n* = 22 and A4 where *n* = 23). Data are presented as median with interquartile range. *p < 0.05

Of all the measured anxiety- and boldness-related parameters, significant differences between the median values of groups were only shown with respect to freezing time (*H*[4] = 20.8, *p* = 0.0003, Fig. 7b) and time spent in the dark compartment of the maze (*H*[4] = 18.0, *p* = 0.001, Fig. 7c).

Specifically, adult fish in the A1 (1.3 s, *p* = 0.03, *d* = 0.5, 95CI [−1.107, 0.070]) and A8 (2.6 s, *p* = 0.02, *d* = 0.7, 95CI [− 1.279, − 0.112]) groups froze significantly less than did the non-stressed fish (37.0 s). Fish in group A8 also spent significantly less time in the dark compartment (0.0 s), compared to their A0 counterparts (104.2 s, *p* = 0.02, *d* = 0.8, 95CI [− 1.342, − 0.169]). Group medians for all other parameters were similar (total distance swam: *H*[4] = 5.3, *p* = 0.256, Fig. 7a; time spent in the border zone: *H*[4] = 5.1, *p* = 0.281, Fig. 7d; time spent in the proximity of the aggressor: *H*[4] = 8.1, *p* = 0.089, Fig. 7f). Although the median times spent in the open field of the maze differed significantly between groups (*H*[4] = 15.0, *p* = 0.005, Fig. 7e), no pairwise differences between non-stressed-exposed and either of the stress-exposed groups were shown. Descriptive statistics are provided in supplementary Tables 4A – F.

Interestingly, zebrafish showed distinct freezing responses in the NTT and the nOFT with control fish spending significantly less time freezing in the NTT, compared to the nOFT (A0: 3.8 s vs 37 s, *p* = 0.0004, *d* = 1.2, 95CI [0.576–1.809]; Table 1; data not represented in figures). Additionally, distinct freezing responses were observed between stress-exposed groups across the two tests, highlighting the differential behavioural responses in the two tests after CAS-paired trauma exposure (A1: 5.2 s vs 1.3 s, *p* = 0.014, *d* = 0.5, 95CI [− 0.079 to 1.123]; A4: 5.9 s vs 53.7 s, *p* = 0.018, *d* = 1.1, 95CI [0.500–1.748]; A8: 6.7 s vs 2.6 s, *p* = 0.026, *d* = 0.3, 95CI [− 0.268 to 0.871]; A12: 4.9 s vs 12.7 s, *p* = 0.17, *d* = 0.9, 95CI [0.259–1.443]; data not represented on figures).

**Table 1:** Descriptive statistics pertaining to time spent freezing by adult fish in the NTT and nOFT

Exposure groups	Descriptive statistics				
NTT vs nOFT	Median time spent freezing (sec)	Mean ± SD	*p*	*d*	CId
A0	3.8 vs 37	3.635 ± 1.619 vs 62.93 ± 69.98	0.0004	1.2	0.576–1.809
A1	5.2 vs 1.3	6.675 ± 4.411 vs 28.89 ± 59.67	0.0141	0.5	− 0.079 to 1.123
A4	5.9 vs 53.7	8.826 ± 6.876 vs 82.78 ± 92.38	0.0177	1.1	0.500–1.748
A8	6.7 vs 2.6	9.451 ± 7.096 vs 20.34 ± 50.31	0.0257	0.3	− 0.268 to 0.871
A12	4.9 vs 12.7	5.897 ± 5.082 vs 52.55 ± 77.01	0.1702	0.9	0.259–1.443

## Discussion

In this work, we explored the impact of trauma-paired CAS concentrations using different numbers of CAS-donating zebrafish on the associated behavioural responses of juvenile and adult zebrafish. Our data revealed four main findings, i.e. (1) stress-exposure, irrespective of CAS concentration as extracted from different CAS-donating zebrafish, had a limited impact on the overall behaviour of juvenile zebrafish; (2) adult stress–exposed fish showed less exploration and more risk-taking behaviour in the NTT and the nOFT; (3) the overall impact of stress in adult zebrafish was modulated by CAS concentration as extracted from different CAS-donating zebrafish and (4) the anxiety-like behaviour of control and stress exposed adult zebrafish is distinctly emulated in the NTT and nOFT, as evidenced by freezing behaviours.

Zebrafish, across all life stages, have emerged as a prominent species for investigating stress and anxiety-related behaviours (Collier et al. 2017; Demin et al. 2021; Egan et al. 2009; Lucon-Xiccato et al. 2020; Wu et al. 2023). They exhibit a heightened sensitivity to diverse stressors including novelty, anxiogenic drug exposure, CAS and predator exposure (Cachat et al. 2011; Collier et al. 2017). Several behavioural paradigms have been proposed to assess and quantify these behaviours, including the LDT, OFT and NTT (Collier et al. 2017; Kysil et al. 2017; Maximino et al. 2010). While CAS has been a subject of study for the past few decades (von Frisch 1938), several uncertainties confound its application in anxiety-related studies. The lack of standardised CAS extraction methods, specifically pertaining to the number of CAS donors used (Maximino et al. 2018; Speedie & Gerlai 2008; Theron et al. 2023), not only introduce methodological variation across studies, but also complicate between-laboratory conclusions relating to stress research in this species. To this end, we aimed to explore how increasing the number of CAS-donating zebrafish when extracting CAS would alter anxiety- and risk-taking behaviours when paired with a standardised traumatic stress protocol in both juvenile and adult zebrafish.

### Behaviour of juvenile zebrafish

In terms of our first main finding, the post-stress responses of juvenile zebrafish in the LDT and OFT were striking. Compared to non-stressed fish, the behaviour of stressed juvenile fish remained unchanged across most of the measured LDT (Fig. 4) and OFT (Fig. 5) parameters. This observation is intriguing considering the severity of the stress protocol applied. One plausible interpretation might be that during stress-sensitive developmental stages, exposure to excessive or traumatic stress may heighten the sensitivity of neuroendocrine responses to stress (Agorastos et al. 2018; Pervanidou & Chrousos 2018). This may result in a twofold outcome: either an exaggerated and prolonged activation or, alternatively, hypoactivation of the stress system, the latter resulting in a vulnerable phenotype marked with disrupted stress reactivity (Agorastos et al. 2018; Pervanidou & Chrousos 2018). Here, our data point to the latter outcome, manifesting as subtle behavioural adaptations, which only reached significance with respect to one of the measured parameters, i.e. thigmotaxis in the LDT (Fig. 4d). Importantly, it should be considered that the effects of early-life stress exposure on behavioural outputs may also only become evident in adulthood, i.e. post-neurodevelopment, as has been shown before (Eachus et al. 2021) and which is supported by the present results (see below).

While unexpected, the behaviour of the non-stressed juvenile cohort needs closer attention to fully explain this result. Anxiety-related assessments in zebrafish involve the evaluation of behavioural responses within an unfamiliar environment (Cachat et al. 2011; Collier et al. 2017; Kysil et al. 2017; Maximino et al. 2010). Exposure to novel and potentially dangerous environments, as was arguably represented here by the LDT and OFT arenas, triggers naturalistic avoidance-related behaviours in various taxa, including zebrafish, a phenomenon believed to originate from their evolutionarily ingrained anti-predator instincts (Blaser & Penalosa 2011; Collier et al. 2017; Levin et al. 2007; Tan et al. 2022). In this sense, a significant reduction in thigmotaxic behaviour is generally regarded as a decreased anxiety-like response (Ahmad & Richardson 2013; Collier et al. 2017; Lockwood et al. 2004; Schnörr et al. 2012; Wu et al. 2023). However, considering the evident reduction in thigmotaxic behaviour of fish in the J1 group, it is likely that this response is related to a stress-induced adaptation to the *naturalistic open field avoidance* shown here to a novel arena by control-exposed fish. Indeed, fish have been shown to deliberately suppress their stress response, potentially resulting in less anxiety-like behaviours (Schreck & Tort 2016). In line with this perspective, we have shown previously, albeit in adult zebrafish, that stress memory consolidation is robustly modulated by the degree of psychobiological stress experienced (Theron et al. 2023). Specifically, zebrafish exposed to a severe stressor (which included CAS exposure) in the presence of additional adrenaline exposure showed less anxiety-like behaviour in an NTT, compared to zebrafish exposed to either the stressor (with CAS) or adrenaline alone. Further, since the anxiety-like behavioural responses of zebrafish exposed to a stressor (with CAS) and adrenaline, respectively, are similar (Theron et al. 2023), it is possible that CAS itself elicits an analogous biological stress response in zebrafish to that of the adrenergic system in isolation. The present result would lend further support to this hypothesis in showing that juvenile zebrafish exposed to CAS extracted from varying numbers of CAS donors showed less anxiety-like behaviour or, alternatively, increased risk-taking behaviour, compared to fish stressed in the same manner, but in the presence of other CAS concentrations. Although not statistically significant, similar noteworthy trends were also mimicked by a decrease in the freezing behaviour of J1-fish, compared to J0 fish, in both the LDT (Fig. 4b) and the OFT (Fig. 5b). It is thus also conceivable that certain behaviours may exhibit greater sensitivity to specific experimental manipulations than others, or alternatively, that some parameters, i.e. thigmotaxis, more robustly identify stress-associated behavioural outcomes. This has also been shown by others (De Marco et al. 2014). Still, that no change in the thigmotaxis of J1 zebrafish was observed in the OFT remains perplexing. A potential explanation for this could be related to habituation, since the dimensions of the LDT and OFT applied in sequence here were similar (Bencan et al. 2009; Wong et al. 2010). Collectively, unanticipated post-stress behavioural responses, as also shown here, are not entirely unknown in zebrafish research. A similar finding was reported by Fontana et al. (2021a, b), who also showed decreased thigmotaxis in juvenile (21-day-old) zebrafish after application of a 7-day stressor. As such, paradoxical responses of juvenile zebrafish in stress-related behavioural studies should be considered as equally important indicators of stress responses.

### Behaviour of adult zebrafish

Adult zebrafish showed a CAS group–specific adaption in terms of their anxiety-like and risk-taking behaviours, compared to fish in the A0 group (Figs. 6 and 7), with CAS extracted from eight zebrafish notably producing the most consistent behavioural change. In the NTT, changes were observed throughout all measured parameters. In the nOFT, findings were less clear, although zebrafish in the A8 group also presented with decreased scototaxis and freezing responses, compared to their A0 counterparts.

In terms of the measured locomotor parameters in the NTT, all stressed zebrafish presented with decreased tank exploration, compared to control-exposed fish (Fig. 6a). This was evinced by a significant reduction in the total distance swam for all groups, except the A1 group, which marginally missed significance (*p* = 0.063). Except for fish in the A1 and A12 groups, the same trend was shown in terms of freezing responses, with increased freezing generally regarded as anxiety-like behaviour (Cachat et al. 2011; Collier et al. 2017; Egan et al. 2009; Kysil et al. 2017; Stewart et al. 2012, 2011; Fig. 6b). This result must be considered together with the increased top-dwelling behaviour, which is normally regarded as an anxiolytic response (Bencan et al. 2009; Collier et al. 2017; Kysil et al. 2017) or alternatively increased risk-taking (Fontana et al. 2021a, b; Thörnqvist et al. 2019) behaviour of the same fish, shown in Fig. 6d. We thus propose that the stress protocol employed here, combined with different concentrations of CAS extracted from up to eight fish, resulted in inflated anxiety-like and risk-taking behaviour, when compared to the responses of A0 zebrafish. Similarly, although differing in their method of extraction and concentration variation, Speedie and Gerlai (2008) showed that increasing CAS concentrations were associated with increased anxiety-like behaviour in an NTT, however only up to a ‘medium’ concentration, whereas higher concentrations elicited behaviours akin to that shown by the control (Speedie & Gerlai 2008). Although following a slightly different approach, Li et al. (2023a, b) showed the same, with the stock solution (CAS extracted from 10 donor fish) causing significantly elevated anxiety-like behaviours, compared to serial dilutions of said stock. Although it is difficult to directly compare our findings with that of previous authors, the present results follow a trend similar to that of Speedie and Gerlai (2008), with CAS extracted from eight fish causing the most noticeable effect on the behavioural response of adult fish, in general (Fig. 6a–c). A possible explanation for these results could be related to the magnitude of the psychobiological stress experienced with differing CAS concentrations, an aspect that we alluded to earlier.

In contrast, findings from the nOFT revealed a notable divergence from those observed in the NTT, particularly in terms of freezing behaviours. Specifically, in the NTT, fish from the A8 group demonstrated a decrease in freezing behaviours, whereas the nOFT revealed an increase in freezing behaviours compared to the controls (Figs. 6b and 7b). Despite potential confounding factors, the behaviour of control-exposed fish highlights an intriguing discovery: they displayed significantly fewer freezing behaviours in the NTT compared to the nOFT (Table 1; *p* = 0.0004). This observation is interesting as it underscores a phenotypic difference in the naturalistic behaviours of zebrafish across the two tests. Zebrafish inhabit natural environments such as rice paddies, shallow ponds and standing water bodies (Kalueff et al., 2014b; Spence et al. 2008). In these environments, zebrafish are exposed to sympatric predators, including fishing birds (Spence et al. 2006), which may trigger escape behaviours. It is highly likely that the shallow depth typical of the nOFT may more closely resemble the natural zebrafish habitat, potentially leading to a greater degree of freezing behaviour. Conversely, the NTT’s greater depth offers a potential escape route from predators, which could result in reduced freezing responses. The notable difference in the etiological meaning of the two tests thus becomes evident in that the NTT simulates an escapable scenario, while the nOFT represents a more exposed environment. As such, freezing behaviours of fish can arguably not be compared directly between the two tests. It should be noted that although CAS undoubtedly influenced the stress responses of zebrafish in a concentration-specific manner, a definite conclusion regarding the most stressogenic concentration remains difficult to draw. While exposure to CAS extracted from eight zebrafish is consistently associated with the most robust presentation of anxiety-like behaviour, neurobiological measurements of among others, noradrenalin, will be needed to corroborate these data.

While our study provides valuable insights, some important limitations of our approach should be noted. Although we did not assess either trauma or the effects of CAS concentration on its own as stress-modulatory factors, this has been investigated extensively before, using among others serial dilutions of stock solutions prepared from one or more fish. Considering the introduction to this paper, this was not the focus of our investigation. However, conducting neurochemical analyses, specifically of cortisol and noradrenaline, would have offered valuable insights into the neurological and physiological mechanisms underpinning the observed behavioural changes, especially as it manifested as a function of varying numbers of CAS donors. In the same sense, it would have been fruitful to quantify and characterize CAS between the different groups to confirm whether actual concentration differences could be observed between the different donor groups. However, while these and other similar studies are on-going, the pertinent question in this work was to establish if methodological variance in so far as the number of CAS-donating zebrafish would influence the behavioural outcomes of trauma-exposed fish. Still, addressing the collective of these limitations in future studies will improve our understanding of the intricate interplay between behaviour and underlying physiological processes.

## Conclusion

Here, we show that CAS, extracted from different numbers of donating zebrafish, is an important factor for consideration in zebrafish stress research. Further, juvenile zebrafish exhibited reduced anxiety-related thigmotactic behaviours in the LDT when exposed to trauma paired with CAS extracted from one adult zebrafish. Conversely, adult zebrafish display a donor number–dependent effect of CAS exposure on anxiety-related outcomes, where stress-paired CAS extracted from eight zebrafish ostensibly produced the most notable anxiety-like response. Several factors may underlie these findings. While the study revealed disparate responses between juvenile and adult zebrafish, it did not specifically establish whether juveniles perceive the same number of CAS-donating zebrafish and by implication concentrations, as aversive as observed in adults. It is plausible that juvenile zebrafish may demonstrate less aversion to CAS exposure than adult fish or demonstrate greater resilience to traumatic stress overall. Nonetheless, our study represents the first investigation into how methodological variance in terms of different numbers of CAS-donating zebrafish paired with traumatic stress modulates the behaviours of both juvenile and adult zebrafish. While our findings are valuable in improving experimental design in zebrafish stress research, the data presented here must be extended on a neurobiological level to clarify the physiological underpinnings of the present results. Furthermore, when employing multiple behavioural paradigms to assess for anxiety-like behaviours in zebrafish, it is crucial to consider the test-specific naturalistic behaviour as an explanatory background against which to interpret experimental results. Collectively, this study establishes CAS as a key modulator of the stress response in zebrafish, shedding light on the intricate interplay between age, number of CAS donors and behavioural outcomes in traumatic stress–related contexts.

## Supplementary Information

Below is the link to the electronic supplementary material.

Supplementary file1 (DOCX 27 KB)

Supplementary file2 (DOCX 24 KB)

Supplementary file3 (DOCX 27 KB)

Supplementary file4 (DOCX 38 KB)

## Data Availability

The data and materials for all experiments are available at Mendeley Data (file designation: CvS_DWW_Zf_CAS_Data_2024; DOI:10.17632/djf5n72cbn.1).

## Declarations

This study was approved by the Animcare Research Ethics Committee of the NWU (approval number: NWU-00754–22-A5; Committee Registration Nr. AREC-130913–015; Approval date: 01 June 2022). All procedures were in accordance with the rules and guidelines stipulated by the South African National Standard 10,386:2021 *“The care and use of animals for scientific purposes”*. Further, all animal experiments complied with the ARRIVE guidelines.

## References

[CR1] Agorastos A, Pervanidou P, Chrousos GP, Kolaitis G (2018) Early life stress and trauma: developmental neuroendocrine aspects of prolonged stress system dysregulation. Hormones 17(4):507–520 30280316 10.1007/s42000-018-0065-x

[CR2] Agorastos A, Pervanidou P, Chrousos GP, Baker DG (2019) Developmental trajectories of early life stress and trauma: a narrative review on neurobiological aspects beyond stress system dysregulation. Front Psych 10:118 10.3389/fpsyt.2019.00118 PMC6421311 30914979

[CR3] Ahmad F, Richardson MK (2013) Exploratory behaviour in the open field test adapted for larval zebrafish: impact of environmental complexity. Behav Process 92:88–98. 10.1016/j.beproc.2012.10.014 10.1016/j.beproc.2012.10.014 23123970

[CR4] Aleström P, D’Angelo L, Midtlyng PJ, Schorderet DF, Schulte-Merker S, Sohm F, Warner S (2020) Zebrafish: housing and husbandry recommendations. Lab Anim 54(3):213–224 31510859 10.1177/0023677219869037 PMC7301644

[CR5] Alsop D, Vijayan M (2009) The zebrafish stress axis: molecular fallout from the teleost-specific genome duplication event. Gen Comp Endocrinol 161(1):62–66 18930731 10.1016/j.ygcen.2008.09.011

[CR6] Ariyomo TO, Carter M, Watt PJ (2013) Heritability of boldness and aggressiveness in the zebrafish. Behav Genet 43:161–167 23354973 10.1007/s10519-013-9585-y

[CR7] Atrooz F, Alkadhi KA, Salim S (2021) Understanding stress: insights from rodent models. Curr Res Neurobiol 2:100013 36246514 10.1016/j.crneur.2021.100013 PMC9559100

[CR8] Bale TL, Abel T, Akil H, Carlezon WA Jr, Moghaddam B, Nestler EJ, Ressler KJ, Thompson SM (2019) The critical importance of basic animal research for neuropsychiatric disorders. Neuropsychopharmacology 44(8):1349–1353 31048727 10.1038/s41386-019-0405-9 PMC6784904

[CR9] Belujon P, Grace AA (2015) Regulation of dopamine system responsivity and its adaptive and pathological response to stress. Proc Royal Society b: Biol Sci 282(1805):20142516 10.1098/rspb.2014.2516 PMC4389605 25788601

[CR10] Bencan Z, Sledge D, Levin ED (2009) Buspirone, chlordiazepoxide and diazepam effects in a zebrafish model of anxiety. Pharmacol Biochem Behav 94(1):75–80 19643124 10.1016/j.pbb.2009.07.009 PMC2771628

[CR11] Blaser R, Penalosa Y (2011) Stimuli affecting zebrafish (Danio rerio) behavior in the light/dark preference test. Physiol Behav 104(5):831–837 21839758 10.1016/j.physbeh.2011.07.029

[CR12] Cachat JM, Canavello PR, Elegante MF, Bartels BK, Elkhayat SI, Hart P C, Tien AK, Tien DH, Beeson E, Mohnot S (2011) Modeling stress and anxiety in zebrafish. Zebrafish models in neurobehavioral research 73–88

[CR13] Chivers DP, Wisenden BD, Hindman CJ, Michalak TA, Kusch RC, Kaminskyj SG, Jack KL, Ferrari MC, Pollock RJ, Halbgewachs CF (2007) Epidermal ‘alarm substance’cells of fishes maintained by non-alarm functions: possible defence against pathogens, parasites and UVB radiation. Proc Royal Soc b: Biol Sci 274(1625):2611–2619 10.1098/rspb.2007.0709 PMC2275884 17686729

[CR14] Chivers DP, Brown GE, Ferrari MC (2012) The evolution of alarm substances and disturbance cues in aquatic animals. Chem Ecol Aquat Syst 127–139

[CR15] Chrousos GP (2009) Stress and disorders of the stress system. Nat Rev Endocrinol 5(7):374–381 19488073 10.1038/nrendo.2009.106

[CR16] Chrousos GP, Gold PW (1992) The concepts of stress and stress system disorders: overview of physical and behavioral homeostasis. JAMA 267(9):1244–1252 1538563

[CR17] Cohen J (1988) Statistical power analysis for the behavioral sciences. Abingdon. In: United Kingdom, Routledge

[CR18] Collier AD, Kalueff AV, Echevarria DJ (2017) Zebrafish models of anxiety-like behaviors. In The rights and wrongs of zebrafish: Behavioral phenotyping of zebrafish, Springer , pp 45–72

[CR19] Compas BE (2006) Psychobiological processes of stress and coping: implications for resilience in children and adolescents—comments on the papers of Romeo & McEwen and Fisher et al. Ann N Y Acad Sci 1094(1):226–234 17347354 10.1196/annals.1376.024

[CR20] Dahlbom SJ, Lagman D, Lundstedt-Enkel K, Sundström LF, Winberg S (2011) Boldness predicts social status in zebrafish (Danio rerio). PLoS ONE 6(8):e23565 21858168 10.1371/journal.pone.0023565 PMC3157393

[CR21] de Abreu MS, Demin KA, Giacomini AC, Amstislavskaya TG, Strekalova T, Maslov GO, Kositsin Y, Petersen EV, Kalueff AV (2021) Understanding how stress responses and stress-related behaviors have evolved in zebrafish and mammals. Neurobiol Stress 15:100405 34722834 10.1016/j.ynstr.2021.100405 PMC8536782

[CR22] De Marco RJ, Groneberg AH, Yeh C-M, Treviño M, Ryu S (2014) The behavior of larval zebrafish reveals stressor-mediated anorexia during early vertebrate development. Front Behav Neurosci 8:367 25368561 10.3389/fnbeh.2014.00367 PMC4202704

[CR23] Demin KA, Taranov AS, Ilyin NP, Lakstygal AM, Volgin AD, de Abreu MS, Strekalova T, Kalueff AV (2021) Understanding neurobehavioral effects of acute and chronic stress in zebrafish. Stress 24(1):1–18 32036720 10.1080/10253890.2020.1724948

[CR24] DeRosse P, Barber AD (2020) Overlapping neurobiological substrates for early life stress and resilience to psychosis. Biol Psychiatry: Cognitive Neurosci Neuroimaging 10.1016/j.bpsc.2020.09.003 PMC7878198 33097471

[CR25] Døving KB, Lastein S (2009) The alarm reaction in fishes—odorants, modulations of responses, neural pathways. Ann N Y Acad Sci 1170(1):413–423 19686169 10.1111/j.1749-6632.2009.04111.x

[CR26] Eachus H, Choi M-K, Ryu S (2021) The effects of early life stress on the brain and behaviour: insights from zebrafish models. Front Cell Dev Biol 1209 10.3389/fcell.2021.657591 PMC8335398 34368117

[CR27] Egan RJ, Bergner CL, Hart PC, Cachat JM, Canavello PR, Elegante MF, Elkhayat SI, Bartels BK, Tien AK, Tien DH (2009) Understanding behavioral and physiological phenotypes of stress and anxiety in zebrafish. Behav Brain Res 205(1):38–44 19540270 10.1016/j.bbr.2009.06.022 PMC2922906

[CR28] Fontana BD, Cleal M, Norton WH, Parker MO (2021a) The impact of chronic unpredictable early-life stress (CUELS) on boldness and stress-reactivity: differential effects of stress duration and context of testing. Physiol Behav 240:113526 34246665 10.1016/j.physbeh.2021.113526

[CR29] Fontana BD, Gibbon AJ, Cleal M, Norton WH, Parker MO (2021b) Chronic unpredictable early-life stress (CUELS) protocol: early-life stress changes anxiety levels of adult zebrafish. Prog Neuropsychopharmacol Biol Psychiatry 108:110087 32889032 10.1016/j.pnpbp.2020.110087

[CR30] Giorgetta C, Grecucci A, Zuanon S, Perini L, Balestrieri M, Bonini N, Sanfey AG, Brambilla P (2012) Reduced risk-taking behavior as a trait feature of anxiety. Emotion 12(6):1373 22775123 10.1037/a0029119

[CR31] Griffiths BB, Schoonheim PJ, Ziv L, Voelker L, Baier H, Gahtan E (2012) A zebrafish model of glucocorticoid resistance shows serotonergic modulation of the stress response. Front Behav Neurosci 6:68 23087630 10.3389/fnbeh.2012.00068 PMC3468897

[CR32] Hare A, Zimmer A, LePabic R, Morgan A, Gilmour K (2021) Early-life stress influences ion balance in developing zebrafish (Danio rerio). J Comp Physiol B 191(1):69–84 33064210 10.1007/s00360-020-01319-9

[CR33] Howe K, Clark MD, Torroja CF, Torrance J, Berthelot C, Muffato M, Collins JE, Humphray S, McLaren K, Matthews L (2013) The zebrafish reference genome sequence and its relationship to the human genome. Nature 496(7446):498–503 23594743 10.1038/nature12111 PMC3703927

[CR34] Jesuthasan S, Krishnan S, Cheng R-K, Mathuru A (2021) Neural correlates of state transitions elicited by a chemosensory danger cue. Prog Neuro-Psychopharmacol Biol Psychiatry 111:110110. 10.1016/j.pnpbp.2020.110110 10.1016/j.pnpbp.2020.110110 32950538

[CR35] Juruena MF, Bourne M, Young AH, Cleare AJ (2021) Hypothalamic-pituitary-adrenal axis dysfunction by early life stress. Neurosci Lett 759:136037 34116195 10.1016/j.neulet.2021.136037

[CR36] Kalueff AV, Gebhardt M, Stewart AM, Cachat JM, Brimmer M, Chawla JS, Craddock C, Kyzar EJ, Roth A, Landsman S (2013) Towards a comprehensive catalog of zebrafish behavior 1.0 and beyond. Zebrafish 10(1):70–86 23590400 10.1089/zeb.2012.0861 PMC3629777

[CR37] Kalueff AV, Echevarria DJ, Stewart AM (2014a) Gaining translational momentum: more zebrafish models for neuroscience research. Prog Neuro-Psychopharmacol Biol Psychiatry 55:1–6. 10.1016/j.pnpbp.2014.01.022 10.1016/j.pnpbp.2014.01.022 24593944

[CR38] Kalueff AV, Stewart AM, Gerlai R (2014b) Zebrafish as an emerging model for studying complex brain disorders. Trends Pharmacol Sci 35(2):63–75. 10.1016/j.tips.2013.12.002 24412421 10.1016/j.tips.2013.12.002 PMC3913794

[CR39] Kysil EV, Meshalkina DA, Frick EE, Echevarria DJ, Rosemberg DB, Maximino C, Lima MG, Abreu MS, Giacomini AC, Barcellos LJ (2017) Comparative analyses of zebrafish anxiety-like behavior using conflict-based novelty tests. Zebrafish 14(3):197–208 28459655 10.1089/zeb.2016.1415

[CR40] Levin ED, Bencan Z, Cerutti DT (2007) Anxiolytic effects of nicotine in zebrafish. Physiol Behav 90(1):54–58 17049956 10.1016/j.physbeh.2006.08.026

[CR41] Li Y, Yan Z, Lin A, Li X, Li K (2023a) Non-dose-dependent relationship between antipredator behavior and conspecific alarm substance in zebrafish. Fishes 8(2):76

[CR42] Li Y, Yan Z, Lin A, Yang X, Li X, Yin X, Li W, Li K (2023) Novel epidermal oxysterols function as alarm substances in zebrafish. bioRxiv 2023.2009. 2026.559639. 10.1016/j.isci.2024.109660 PMC11033690 38650983

[CR43] Lima MG, do Santos Silva SDN, do Carmo Silva RX, Oliveira KRHM, Batista EDJO, Maximino C, Herculano AM (2015) Putative involvement of the nitrergic system on the consolidation, but not initiation, of behavioral sensitization after conspecific alarm substance in zebrafish. Pharmacol Biochem Behav 139:127–133 26257339 10.1016/j.pbb.2015.08.005

[CR44] Lima MG, dos Carmo Silva RX, dos Santos Silva SDN, dos Santos Rodrigues LDS, Oliveira KRHM, Batista EDJO, Maximino C, Herculano AM (2016) Time-dependent sensitization of stress responses in zebrafish: a putative model for post-traumatic stress disorder. Behav Process 128:70–82 10.1016/j.beproc.2016.04.009 27102763

[CR45] Lockwood B, Bjerke S, Kobayashi K, Guo S (2004) Acute effects of alcohol on larval zebrafish: a genetic system for large-scale screening. Pharmacol Biochem Behav 77(3):647–654 15006478 10.1016/j.pbb.2004.01.003

[CR46] Lopez-Luna J, Al-Jubouri Q, Al-Nuaimy W, Sneddon LU (2017) Impact of stress, fear and anxiety on the nociceptive responses of larval zebrafish. PLoS ONE 12(8):e0181010 28767661 10.1371/journal.pone.0181010 PMC5540279

[CR47] Lucon-Xiccato T, Di Mauro G, Bisazza A, Bertolucci C (2020) Alarm cue-mediated response and learning in zebrafish larvae. Behav Brain Res 380:112446 31870779 10.1016/j.bbr.2019.112446

[CR48] Madaro A, Kristiansen TS, Pavlidis MA (2020) How fish cope with stress? The welfare of fish 251–281

[CR49] Masuda M, Ihara S, Mori N, Koide T, Miyasaka N, Wakisaka N, Yoshikawa K, Watanabe H, Touhara K, Yoshihara Y (2024) Identification of olfactory alarm substances in zebrafish. Curr Biol 34(7):1377–1389 38423017 10.1016/j.cub.2024.02.003

[CR50] Maximino C, de Brito TM, da Silva Batista AW, Herculano AM, Morato S, Gouveia A Jr (2010) Measuring anxiety in zebrafish: a critical review. Behav Brain Res 214(2):157–171 20510300 10.1016/j.bbr.2010.05.031

[CR51] Maximino C, Silva RX, da Silva S, Rodrigues LS, Barbosa H, de Carvalho TS, Leão LKR, Lima MG, Oliveira KRM, Herculano AM (2015) Non-mammalian models in behavioral neuroscience: consequences for biological psychiatry. Front Behav Neurosci 9:233 26441567 10.3389/fnbeh.2015.00233 PMC4561806

[CR52] Maximino C, Meinerz DL, Fontana BD, Mezzomo NJ, Stefanello FV, Prestes ADS, Batista CB, Rubin MA, Barbosa NV, Rocha JBT (2018) Extending the analysis of zebrafish behavioral endophenotypes for modeling psychiatric disorders: fear conditioning to conspecific alarm response. Behav Process 149:35–42 10.1016/j.beproc.2018.01.020 29409977

[CR53] Maximino C, do Carmo Silva RX, dos Santos Campos K, de Oliveira JS, Rocha SP, Pyterson MP, dos Santos Souza DP, Feitosa LM, Ikeda SR, Pimentel AF (2019) Sensory ecology of ostariophysan alarm substances. J Fish Biology 95(1):274–286 30345536 10.1111/jfb.13844

[CR54] Molet J, Maras PM, Avishai-Eliner S, Baram TZ (2014) Naturalistic rodent models of chronic early-life stress. Dev Psychobiol 56(8):1675–1688 24910169 10.1002/dev.21230 PMC4777289

[CR55] Musazzi L, Tornese P, Sala N, Popoli M (2017) Acute or chronic? A Stressful Question. Trends Neurosci 40(9):525–535 28778394 10.1016/j.tins.2017.07.002

[CR56] Nathan FM, Ogawa S, Parhar IS (2015) Neuronal connectivity between habenular glutamate-kisspeptin1 co-expressing neurons and the raphe 5-HT system. J Neurochem 135(4):814–829 26250886 10.1111/jnc.13273 PMC5049628

[CR57] Nestler EJ, Hyman SE (2010) Animal models of neuropsychiatric disorders. Nat Neurosci 13(10):1161 20877280 10.1038/nn.2647 PMC3750731

[CR58] Oliveira TA, Koakoski G, da Motta AC, Piato AL, Barreto RE, Volpato GL, Barcellos LJG (2014) Death-associated odors induce stress in zebrafish. Horm Behav 65(4):340–344 24613177 10.1016/j.yhbeh.2014.02.009

[CR59] Pechtel P, Pizzagalli DA (2011) Effects of early life stress on cognitive and affective function: an integrated review of human literature. Psychopharmacology 214(1):55–70 20865251 10.1007/s00213-010-2009-2 PMC3050094

[CR60] Pervanidou P, Chrousos GP (2018) Early-life stress: from neuroendocrine mechanisms to stress-related disorders. Hormone Res Paediatr 89(5):372–379 10.1159/000488468 29886495

[CR61] Piato ÂL, Capiotti KM, Tamborski AR, Oses JP, Barcellos LJ, Bogo MR, Lara DR, Vianna MR, Bonan CD (2011) Unpredictable chronic stress model in zebrafish (Danio rerio): behavioral and physiological responses. Prog Neuropsychopharmacol Biol Psychiatry 35(2):561–567 21187119 10.1016/j.pnpbp.2010.12.018

[CR62] Quadros VA, Silveira A, Giuliani GS, Didonet F, Silveira AS, Nunes ME, Silva TO, Loro VL, Rosemberg DB (2016) Strain-and context-dependent behavioural responses of acute alarm substance exposure in zebrafish. Behav Proc 122:1–11 10.1016/j.beproc.2015.10.014 26524408

[CR63] Ramsay JM, Feist GW, Varga ZM, Westerfield M, Kent ML, Schreck CB (2006) Whole-body cortisol is an indicator of crowding stress in adult zebrafish. Danio Rerio Aquaculture 258(1–4):565–574

[CR64] Ramsay JM, Feist GW, Varga ZM, Westerfield M, Kent ML, Schreck CB (2009) Whole-body cortisol response of zebrafish to acute net handling stress. Aquaculture 297(1–4):157–162 25587201 10.1016/j.aquaculture.2009.08.035 PMC4289633

[CR65] Sabadin GR, Biasuz E, Canzian J, Adedara IA, Rosemberg DB (2022) A novel behavioral paradigm to measure anxiety-like behaviors in zebrafish by the concomitant assessment of geotaxis and scototaxis. Prog Neuropsychopharmacol Biol Psychiatry 118:110579 35618149 10.1016/j.pnpbp.2022.110579

[CR66] Schiavone S, Colaianna M, Curtis L (2015) Impact of early life stress on the pathogenesis of mental disorders: relation to brain oxidative stress. Curr Pharm des 21(11):1404–1412 25564385 10.2174/1381612821666150105143358

[CR67] Schnörr S, Steenbergen P, Richardson M, Champagne D (2012) Measuring thigmotaxis in larval zebrafish. Behav Brain Res 228(2):367–374 22197677 10.1016/j.bbr.2011.12.016

[CR68] Schreck CB, Tort L (2016) The concept of stress in fish. In Fish physiology, Elsevier, 35, pp 1–34

[CR69] Silberman DM, Acosta GB, Zorrilla Zubilete MA (2016) Long-term effects of early life stress exposure: role of epigenetic mechanisms. Pharmacol Res 109:64–73. 10.1016/j.phrs.2015.12.033 26774789 10.1016/j.phrs.2015.12.033

[CR70] Simonetti RB, Santos Marques L, Streit DP Jr, Oberst R (2016) Zebrafish (Danio rerio): ethics in animal experimentation. IOSR J Agric Vet Sci Ver I 9(7):2319–2372

[CR71] Smith AR, Ebert EE, Broman-Fulks JJ (2016) The relationship between anxiety and risk taking is moderated by ambiguity. Personality Individ Differ 95:40–44

[CR72] Song C, Liu B-P, Zhang Y-P, Peng Z, Wang J, Collier AD, Echevarria DJ, Savelieva KV, Lawrence RF, Rex CS (2018) Modeling consequences of prolonged strong unpredictable stress in zebrafish: complex effects on behavior and physiology. Prog Neuropsychopharmacol Biol Psychiatry 81:384–394 28847526 10.1016/j.pnpbp.2017.08.021

[CR73] Speedie N, Gerlai R (2008) Alarm substance induced behavioral responses in zebrafish (Danio rerio). Behav Brain Res 188(1):168–177 18054804 10.1016/j.bbr.2007.10.031 PMC2715551

[CR74] Spence R, Fatema M, Reichard M, Huq K, Wahab M, Ahmed Z, Smith C (2006) The distribution and habitat preferences of the zebrafish in Bangladesh. J Fish Biol 69(5):1435–1448

[CR75] Spence R, Gerlach G, Lawrence C, Smith C (2008) The behaviour and ecology of the zebrafish. Danio Rerio Biological Reviews 83(1):13–34 18093234 10.1111/j.1469-185X.2007.00030.x

[CR76] Steenbergen PJ, Richardson MK, Champagne DL (2011) The use of the zebrafish model in stress research. Prog Neuropsychopharmacol Biol Psychiatry 35(6):1432–1451 20971150 10.1016/j.pnpbp.2010.10.010

[CR77] Stewart A, Wu N, Cachat J, Hart P, Gaikwad S, Wong K, Utterback E, Gilder T, Kyzar E, Newman A (2011) Pharmacological modulation of anxiety-like phenotypes in adult zebrafish behavioral models. Prog Neuropsychopharmacol Biol Psychiatry 35(6):1421–1431 21122812 10.1016/j.pnpbp.2010.11.035

[CR78] Stewart A, Gaikwad S, Kyzar E, Green J, Roth A, Kalueff AV (2012) Modeling anxiety using adult zebrafish: a conceptual review. Neuropharmacology 62(1):135–143 21843537 10.1016/j.neuropharm.2011.07.037 PMC3195883

[CR79] Stewart AM, Braubach O, Spitsbergen J, Gerlai R, Kalueff AV (2014) Zebrafish models for translational neuroscience research: from tank to bedside. Trends Neurosci 37(5):264–278. 10.1016/j.tins.2014.02.011 24726051 10.1016/j.tins.2014.02.011 PMC4039217

[CR80] Tan JXM, Ang RJW, Wee CL (2022) Larval zebrafish as a model for mechanistic discovery in mental health. Front Mol Neurosci 15:900213 35813062 10.3389/fnmol.2022.900213 PMC9263853

[CR81] Theron V, Harvey BH, Botha T, Weinshenker D, Wolmarans DW (2023) Life-threatening, high-intensity trauma-and context-dependent anxiety in zebrafish and its modulation by epinephrine. Horm Behav 153:105376 37244195 10.1016/j.yhbeh.2023.105376

[CR82] Thörnqvist P-O, McCarrick S, Ericsson M, Roman E, Winberg S (2019) Bold zebrafish (Danio rerio) express higher levels of delta opioid and dopamine D2 receptors in the brain compared to shy fish. Behav Brain Res 359:927–934 29935279 10.1016/j.bbr.2018.06.017

[CR83] van der Westhuizen C, Botha TL, Finger-Baier K, de Brouwer G, Wolmarans DW (2023) Contingency learning in zebrafish exposed to apomorphine-and levetiracetam. Behav Pharmacol 34(7):424–436 37578419 10.1097/FBP.0000000000000750

[CR84] Van Staden C, Weinshenker D, Finger-Baier K, Botha TL, Brand L, Wolmarans DW (2025) Posttraumatic anxiety-like behaviour in zebrafish is dose-dependently attenuated by the alpha-2A receptor agonist, guanfacine. Behav Pharmacol 36(1):47–59 39718044 10.1097/FBP.0000000000000808

[CR85] Verbitsky A, Dopfel D, Zhang N (2020) Rodent models of post-traumatic stress disorder: behavioral assessment. Transl Psychiatry 10(1):132. 10.1038/s41398-020-0806-x 32376819 10.1038/s41398-020-0806-x PMC7203017

[CR86] von Frisch K (1938) Zur psychologie des fisch-schwarmes. Naturwissenschaften

[CR87] Wisenden BD, Sailer CD, Radenic SJ, Sutrisno R (2011) Maternal inheritance and exploratory-boldness behavioural syndrome in zebrafish. Behaviour 1443–1456

[CR88] Wong K, Elegante M, Bartels B, Elkhayat S, Tien D, Roy S, Goodspeed J, Suciu C, Tan J, Grimes C (2010) Analyzing habituation responses to novelty in zebrafish (Danio rerio). Behav Brain Res 208(2):450–457 20035794 10.1016/j.bbr.2009.12.023

[CR89] Wright D, Rimmer LB, Pritchard VL, Butlin R, Krause J (2003) Inter and intra-population variation in shoaling and boldness in the zebrafish (Danio rerio). J Fish Biol 63:258–259 10.1007/s00114-003-0443-2 12955228

[CR90] Wu J, Yan B, Bao M, Shen J, Zheng P, Wu D, Wang J, Li Z, Jiang K (2023) Early life exposure to chronic unpredictable stress induces anxiety-like behaviors and increases the excitability of cerebellar neurons in zebrafish. Behav Brain Res 437:114160 36257559 10.1016/j.bbr.2022.114160

