# Supplementary material for: The number of conspecific alarm substance donors notably influences the behavioural responses of zebrafish subjected to a traumatic stress procedure
Source: Fish Physiol Biochem. 2025 Feb 26;51(2):55. doi: 10.1007/s10695-025-01468-0 (PMC11865224; doi:10.1007/s10695-025-01468-0)
Supplement: Supplementary file 1 — Supplementary file1 (DOCX 27 KB) [file 10695_2025_1468_MOESM1_ESM.docx]

**The number of conspecific alarm substance donors notably influences the behavioural responses of zebrafish subjected to a traumatic stress procedure**

**Journal: Fish Physiology and Biochemistry**

**C van Staden^a^, K Finger-Baier^b^, D Weinshenker^c^, TL Botha^d^, L Brand^a^, D Wolmarans^a,^***

*^a^Centre of Excellence for Pharmaceutical Sciences, Department of Pharmacology, North-West University, 11 Hoffman Street, Potchefstroom, 2520, South Africa*

*^b^Department Genes - Circuits - Behavior, Max Planck Institute for Biological Intelligence, Martinsried, Germany*

*^c^Department of Human Genetics, Emory University School of Medicine, 615 Michael St., Whitehead 301, Atlanta, GA 30322, USA*

*^d^Department of Zoology, University of Johannesburg, Auckland Park, Johannesburg, 2006, South Africa*

Address correspondence to: De Wet Wolmarans, Center of Excellence for Pharmaceutical Sciences, Faculty of Health Sciences, North-West University, 11 Hoffman Street, Potchefstroom, South Africa.

Email: dewet.wolmarans@nwu.ac.za Telephone: +27 (0) 18 299 2230

**Table 1 – Descriptive statistics pertaining to behaviour of juvenile fish in the LDT**

1. **Locomotor Activity**

| Exposure groups | | Descriptive statistics | | | |
| --- | --- | --- | --- | --- | --- |
|  | | ***Mean ± SD*** | ***p*** | ***d*** | **CI*d*** |
| J0 vs. | **J1** | 625.5 ± 147.5 vs 643.3 ± 107.1 | >0.999 | 0.1 | -0.550 – 0.660 |
|  | **J4** | 625.5 ± 147.5 vs 675.6 ± 140.0 | >0.999 | 0.2 | -0.400 – 0.814 |
|  | **J8** | 625.5 ± 147.5 vs 609.4 ± 135.1 | >0.999 | 0.5 | -1.084 – 0.144 |
|  | **J12** | 625.5 ± 147.5 vs 574.8 ± 167.4 | >0.999 | 0.4 | -1.050 – 0.190 |
| J1 vs. | **J4** | 643.3 ± 107.1 vs 675.6 ± 140.0 | >0.999 | 0.2 | -0.425 – 0.788 |
|  | **J8** | 643.3 ± 107.1 vs 609.4 ± 135.1 | >0.999 | 0.6 | -1.261 – -0.019 |
|  | **J12** | 643.3 ± 107.1 vs 574.8 ± 167.4 | 0.512 | 0.5 | -1.165 – 0.083 |
| J4 vs. | **J8** | 675.6 ± 140.0 vs 609.4 ± 135.1 | >0.999 | **0.8** | -1.403 – -0.146 |
|  | **J12** | 675.6 ± 140.0 vs 574.8 ± 167.4 | 0.140 | 0.7 | -1.293 – -0.033 |
| J8 vs. | **J12** | 609.4 ± 135.1 vs 574.8 ± 167.4 | >0.999 | 0.1 | -0.663 – 0.562 |
| Main effect: *H*(4) = 7.33. *p* = 0.12 | | | | | |

1. **Time Spent Freezing**

| Exposure groups | | Descriptive statistics | | | |
| --- | --- | --- | --- | --- | --- |
|  | | ***Mean ± SD*** | ***p*** | ***d*** | **CI*d*** |
| J0 vs. | **J1** | 34.47 ± 17.83 vs 28.71 ± 11.30 | >0.999 | 0.4 | -0.955 – 0.188 |
|  | **J4** | 34.47 ± 17.83 vs 28.01 ± 14.85 | >0.999 | 0.4 | -0.963 – 0.180 |
|  | **J8** | 34.47 ± 17.83 vs 34.57 ± 19.33 | >0.999 | 0 | -0.561 – 0.571 |
|  | **J12** | 34.47 ± 17.83 vs 43.87 ± 26.48 | >0.999 | 0.4 | -0.162 – 0.994 |
| J1 vs. | **J4** | 28.71 ± 11.30 vs 28.01 ± 14.85 | >0.999 | 0.1 | -0.619 – 0.513 |
|  | **J8** | 28.71 ± 11.30 vs 34.57 ± 19.33 | >0.999 | 0.4 | -0.203 – 0.939 |
|  | **J12** | 28.71 ± 11.30 vs 43.87 ± 26.48 | 0.665 | 0.6 | 0.154 – 1.339 |
| J4 vs. | **J8** | 28.01 ± 14.85 vs 34.57 ± 19.33 | >0.999 | 0.4 | -0.193 – 0.950 |
|  | **J12** | 28.01 ± 14.85 vs 43.87 ± 26.48 | 0.150 | 0.7 | 0.147 – 1.332 |
| J8 vs. | **J12** | 34.57 ± 19.33 vs 43.87 ± 26.48 | >0.999 | 0.4 | -0.177 – 0.978 |
| Main effect: *H*(4) = 6.58. *p* = 0.160 | | | | | |

1. **Total Dark Time**

| Exposure groups | | Descriptive statistics | | | |
| --- | --- | --- | --- | --- | --- |
|  | | ***Mean ± SD*** | ***p*** | ***d*** | **CI*d*** |
| J0 vs. | **J1** | 233.1 ± 115.3 vs 217.9 ± 103.8 | >0.999 | 0.1 | -0.705 – 0.428 |
|  | **J4** | 233.1 ± 115.3 vs 208.5 ± 128.8 | >0.999 | 0.2 | -0.767 – 0.367 |
|  | **J8** | 233.1 ± 115.3 vs 233.6 ± 107.7 | >0.999 | 0 | -0.562 – 0.570 |
|  | **J12** | 233.1 ± 115.3 vs 250.3 ± 82.21 | >0.999 | 0.2 | -0.403 – 0.743 |
| J1 vs. | **J4** | 217.9 ± 103.8 vs 208.5 ± 128.8 | >0.999 | 0.1 | -0.645 – 0.487 |
|  | **J8** | 217.9 ± 103.8 vs 233.6 ± 107.7 | >0.999 | 0.1 | -0.419 – 0.714 |
|  | **J12** | 217.9 ± 103.8 vs 250.3 ± 82.21 | >0.999 | 0.3 | -0.232 – 0.920 |
| J4 vs. | **J8** | 208.5 ± 128.8 vs 233.6 ± 107.7 | >0.999 | 0.2 | -0.358 – 0.777 |
|  | **J12** | 208.5 ± 128.8 vs 250.3 ± 82.21 | >0.999 | 0.4 | -0.195 – 0.960 |
| J8 vs. | **J12** | 233.6 ± 107.7 vs 250.3 ± 82.21 | >0.999 | 0.2 | -0.400 – 0.746 |
| Main effect: *H*(4) = 1.44. *p* = 0.838 | | | | | |

1. **Total Border Time**

| Exposure groups | | Descriptive statistics | | | |
| --- | --- | --- | --- | --- | --- |
|  | | ***Mean ± SD*** | ***p*** | ***d*** | **CI*d*** |
| J0 vs. | **J1** | 266.3 ± 70.27 vs 200.2 ± 85.28 | **0.011** | **0.8** | -1.432 – -0.250 |
|  | **J4** | 266.3 ± 70.27 vs 235.4 ± 68.85 | 0.527 | 0.4 | -1.014 – 0.132 |
|  | **J8** | 266.3 ± 70.27 vs 246.6 ± 79.38 | >0.999 | 0.3 | -0.829 – 0.307 |
|  | **J12** | 266.3 ± 70.27 vs 253.8 ± 58.94 | >0.999 | 0.2 | -0.764 – 0.382 |
| J1 vs. | **J4** | 200.2 ± 85.28 vs 235.4 ± 68.85 | >0.999 | 0.5 | -0.122 – 1.025 |
|  | **J8** | 200.2 ± 85.28 vs 246.6 ± 79.38 | 0.335 | 0.6 | -0.018 – 1.137 |
|  | **J12** | 200.2 ± 85.28 vs 253.8 ± 58.94 | 0.269 | 0.7 | 0.133 – 1.315 |
| J4 vs. | **J8** | 235.4 ± 68.85 vs 246.6 ± 79.38 | >0.999 | 0.2 | -0.417 – 0.716 |
|  | **J12** | 235.4 ± 68.85 vs 253.8 ± 58.94 | >0.999 | 0.3 | -0.290 – 0.859 |
| J8 vs. | **J12** | 246.6 ± 79.38 vs 253.8 ± 58.94 | >0.999 | 0.1 | -0.470 – 0.674 |
| Main effect: *H*(4) = 11.79. *p* = 0.019* | | | | | |
