# Supplementary material for: The number of conspecific alarm substance donors notably influences the behavioural responses of zebrafish subjected to a traumatic stress procedure
Source: Fish Physiol Biochem. 2025 Feb 26;51(2):55. doi: 10.1007/s10695-025-01468-0 (PMC11865224; doi:10.1007/s10695-025-01468-0)
Supplement: Supplementary file 2 — Supplementary file2 (DOCX 24 KB) [file 10695_2025_1468_MOESM2_ESM.docx]

**The number of conspecific alarm substance donors notably influences the behavioural responses of zebrafish subjected to a traumatic stress procedure**

**Journal: Fish Physiology and Biochemistry**

**C van Staden^a^, K Finger-Baier^b^, D Weinshenker^c^, TL Botha^d^, L Brand^a^, D Wolmarans^a,^***

*^a^Centre of Excellence for Pharmaceutical Sciences, Department of Pharmacology, North-West University, 11 Hoffman Street, Potchefstroom, 2520, South Africa*

*^b^Department Genes - Circuits - Behavior, Max Planck Institute for Biological Intelligence, Martinsried, Germany*

*^c^Department of Human Genetics, Emory University School of Medicine, 615 Michael St., Whitehead 301, Atlanta, GA 30322, USA*

*^d^Department of Zoology, University of Johannesburg, Auckland Park, Johannesburg, 2006, South Africa*

Address correspondence to: De Wet Wolmarans, Center of Excellence for Pharmaceutical Sciences, Faculty of Health Sciences, North-West University, 11 Hoffman Street, Potchefstroom, South Africa.

Email: dewet.wolmarans@nwu.ac.za Telephone: +27 (0) 18 299 2230

**Table 2 – Descriptive statistics pertaining to behaviour of juvenile fish in the OFT**

1. **Locomotor Activity**

| Exposure groups | | Descriptive statistics | | | |
| --- | --- | --- | --- | --- | --- |
|  | | ***Mean ± SD*** | ***p*** | ***d*** | **CI*d*** |
| J0 vs. | **J1** | 656.8 ± 224.8 vs 670.9 ± 147.7 | >0.999 | 0.1 | -0.492 – 0.640 |
|  | **J4** | 656.8 ± 224.8 vs 688.5 ± 147.4 | >0.999 | 0.2 | -0.401 – 0.733 |
|  | **J8** | 656.8 ± 224.8 vs 598.4 ± 120.1 | >0.999 | 0.3 | -0.892 – 0.247 |
|  | **J12** | 656.8 ± 224.8 vs 625.9 ± 145.5 | >0.999 | 0.2 | -0.734 – 0.411 |
| J1 vs. | **J4** | 670.9 ± 147.7 vs 688.5 ± 147.4 | >0.999 | 0.1 | -0.448 – 0.685 |
|  | **J8** | 670.9 ± 147.7 vs 598.4 ± 120.1 | 0.992 | 0.5 | -1.112 – 0.041 |
|  | **J12** | 670.9 ± 147.7 vs 625.9 ± 145.5 | >0.999 | 0.3 | -0.881 – 0.270 |
| J4 vs. | **J8** | 688.5 ± 147.4 vs 598.4 ± 120.1 | 0.416 | 0.7 | -1.249 – -0.084 |
|  | **J12** | 688.5 ± 147.4 vs 625.9 ± 145.5 | >0.999 | 0.4 | -1.004 – 0.154 |
| J8 vs. | **J12** | 598.4 ± 120.1 vs 625.9 ± 145.5 | >0.999 | 0.2 | -0.368 – 0.779 |
| Main effect: *H*(4) = 5.48, *p* = 0.241 | | | | | |

1. **Time Spent Freezing**

| Exposure groups | | Descriptive statistics | | | |
| --- | --- | --- | --- | --- | --- |
|  | | ***Mean ± SD*** | ***p*** | ***d*** | **CI*d*** |
| J0 vs. | **J1** | 28.23 ± 15.10 vs 24.28 ± 15.63 | >0.999 | 0.3 | -0.824 – 0.313 |
|  | **J4** | 28.23 ± 15.10 vs 25.92 ± 18.29 | >0.999 | 0.1 | -0.703 – 0.430 |
|  | **J8** | 28.23 ± 15.10 vs 30.05 ± 12.77 | >0.999 | 0.1 | -0.437 – 0.696 |
|  | **J12** | 28.23 ± 15.10 vs 33.75 ± 15.41 | >0.999 | 0.4 | -0.217 – 0.937 |
| J1 vs. | **J4** | 24.28 ± 15.63 vs 25.92 ± 18.29 | >0.999 | 0.1 | -0.470 – 0.662 |
|  | **J8** | 24.28 ± 15.63 vs 30.05 ± 12.77 | 0.483 | 0.4 | -0.170 – 0.974 |
|  | **J12** | 24.28 ± 15.63 vs 33.75 ± 15.41 | 0.115 | 0.6 | 0.021 – 1.193 |
| J4 vs. | **J8** | 25.92 ± 18.29 vs 30.05 ± 12.77 | 0.792 | 0.3 | -0.308 – 0.828 |
|  | **J12** | 25.92 ± 18.29 vs 33.75 ± 15.41 | 0.209 | 0.5 | -0.120 – 1.039 |
| J8 vs. | **J12** | 30.05 ± 12.77 vs 33.75 ± 15.41 | >0.999 | 0.3 | -0.313 – 0.836 |
| Main effect: *H*(4) = 9.52, *p* = 0.049* | | | | | |

1. **Total OF Border Time**

| Exposure groups | | Descriptive statistics | | | |
| --- | --- | --- | --- | --- | --- |
|  | | ***Mean ± SD*** | ***p*** | ***d*** | **CI*d*** |
| J0 vs. | **J1** | 255.7 ± 61.23 vs 234.3 ± 86.31 | >0.999 | 0.3 | -0.853 – 0.285 |
|  | **J4** | 255.7 ± 61.23 vs 257.2 ± 61.54 | >0.999 | 0.03 | -0.541 – 0.590 |
|  | **J8** | 255.7 ± 61.23 vs 242.7 ± 72.33 | >0.999 | 0.2 | -0.761 – 0.374 |
|  | **J12** | 255.7 ± 61.23 vs 256.4 ± 74.51 | >0.999 | 0.01 | -0.562 – 0.582 |
| J1 vs. | **J4** | 234.3 ± 86.31 vs 257.2 ± 61.54 | >0.999 | 0.3 | -0.266 – 0.873 |
|  | **J8** | 234.3 ± 86.31 vs 242.7 ± 72.33 | >0.999 | 0.1 | -0.462 – 0.670 |
|  | **J12** | 234.3 ± 86.31 vs 256.4 ± 74.51 | >0.999 | 0.3 | -0.303 – 0.847 |
| J4 vs. | **J8** | 257.2 ± 61.54 vs 242.7 ± 72.33 | >0.999 | 0.2 | -0.783 – 0.352 |
|  | **J12** | 257.2 ± 61.54 vs 256.4 ± 74.51 | >0.999 | 0.01 | -0.584 – 0.560 |
| J8 vs. | **J12** | 242.7 ± 72.33 vs 256.4 ± 74.51 | >0.999 | 0.2 | -0.387 – 0.759 |
| Main effect: *H*(4) = 1.20, *p* = 0.878 | | | | | |
