# Supplementary material for: The number of conspecific alarm substance donors notably influences the behavioural responses of zebrafish subjected to a traumatic stress procedure
Source: Fish Physiol Biochem. 2025 Feb 26;51(2):55. doi: 10.1007/s10695-025-01468-0 (PMC11865224; doi:10.1007/s10695-025-01468-0)
Supplement: Supplementary file 3 — Supplementary file3 (DOCX 27 KB) [file 10695_2025_1468_MOESM3_ESM.docx]

**The number of conspecific alarm substance donors notably influences the behavioural responses of zebrafish subjected to a traumatic stress procedure**

**Journal: Fish Physiology and Biochemistry**

**C van Staden^a^, K Finger-Baier^b^, D Weinshenker^c^, TL Botha^d^, L Brand^a^, D Wolmarans^a,^***

*^a^Centre of Excellence for Pharmaceutical Sciences, Department of Pharmacology, North-West University, 11 Hoffman Street, Potchefstroom, 2520, South Africa*

*^b^Department Genes - Circuits - Behavior, Max Planck Institute for Biological Intelligence, Martinsried, Germany*

*^c^Department of Human Genetics, Emory University School of Medicine, 615 Michael St., Whitehead 301, Atlanta, GA 30322, USA*

*^d^Department of Zoology, University of Johannesburg, Auckland Park, Johannesburg, 2006, South Africa*

Address correspondence to: De Wet Wolmarans, Center of Excellence for Pharmaceutical Sciences, Faculty of Health Sciences, North-West University, 11 Hoffman Street, Potchefstroom, South Africa.

Email: dewet.wolmarans@nwu.ac.za Telephone: +27 (0) 18 299 2230

**Table 3 – Descriptive statistics pertaining to behaviour of adult fish**

1. **Locomotor Activity**

| Exposure groups | | Descriptive statistics | | | |
| --- | --- | --- | --- | --- | --- |
|  | | ***Mean ± SD*** | ***p*** | ***d*** | **CI*d*** |
| A0 vs. | **A1** | 1876 ± 380.3 vs 1498 ± 298.5 | 0.063 | **1.1** | -1.717 – -0.473 |
|  | **A4** | 1876 ± 380.3 vs 1374 ± 334.9 | **0.0001** | **1.4** | -2.033 – -0.752 |
|  | **A8** | 1876 ± 380.3 vs 1264 ± 220.2 | **<0.0001** | **2.0** | -2.658 – -1.270 |
|  | **A12** | 1876 ± 380.3 vs 1503 ± 218.7 | **0.014** | **1.2** | -1.814 – -0.581 |
| A1 vs. | **A4** | 1498 ± 298.5 vs 1374 ± 334.9 | >0.999 | 0.4 | -0.978 – 0.203 |
|  | **A8** | 1498 ± 298.5 vs 1264 ± 220.2 | **0.033** | **0.9** | -1.503 – -0.287 |
|  | **A12** | 1498 ± 298.5 vs 1503 ± 218.7 | >0.999 | 0.02 | -0.559 – 0.598 |
| A4 vs. | **A8** | 1374 ± 334.9 vs 1264 ± 220.2 | >0.999 | 0.4 | -0.967 – 0.188 |
|  | **A12** | 1374 ± 334.9 vs 1503 ± 218.7 | >0.999 | 0.5 | -0.124 – 1.035 |
| A8 vs. | **A12** | 1503 ± 218.7 vs 1264 ± 220.2 | 0.093 | **1.1** | 0.478 – 1.694 |
| Main effect: *H*(4) = 36.91, *p* = <0.0001**** | | | | | |

1. **Time Spent Freezing**

| Exposure groups | | Descriptive statistics | | | |
| --- | --- | --- | --- | --- | --- |
|  | | ***Mean ± SD*** | ***p*** | ***d*** | **CI*d*** |
| A0 vs. | **A1** | 3.635 ± 1.619 vs 6.675 ± 4.411 | 0.056 | **0.9** | 0.316 – 1.537 |
|  | **A4** | 3.635 ± 1.619 vs 8.826 ± 6.876 | **0.0007** | **1.1** | 0.433 – 1.656 |
|  | **A8** | 3.635 ± 1.619 vs 9.451 ± 7.096 | **<0.0001** | **1.1** | 0.514 – 1.736 |
|  | **A12** | 3.635 ± 1.619 vs 5.897 ± 5.082 | 0.531 | 0.6 | 0.018 – 1.176 |
| A1 vs. | **A4** | 6.675 ± 4.411 vs 8.826 ± 6.876 | >0.999 | 0.4 | -0.221 – 0.958 |
|  | **A8** | 6.675 ± 4.411 vs 9.451 ± 7.096 | 0.1996 | 0.5 | -0.124 – 1.049 |
|  | **A12** | 6.675 ± 4.411 vs 5.897 ± 5.082 | >0.999 | 0.2 | -0.742 – 0.417 |
| A4 vs. | **A8** | 8.826 ± 6.876 vs 9.451 ± 7.096 | >0.999 | 0.1 | -0.483 – 0.661 |
|  | **A12** | 8.826 ± 6.876 vs 5.897 ± 5.082 | 0.389 | 0.5 | -1.064 – 0.097 |
| A8 vs. | **A12** | 9.451 ± 7.096 vs 5.897 ± 5.082 | **0.010** | 0.6 | -1.151 – 0.005 |
| Main effect: *H*(4) = 31.69, *p* = <0.0001**** | | | | | |

1. **Entries Into Top Zone**

| Exposure groups | | Descriptive statistics | | | |
| --- | --- | --- | --- | --- | --- |
|  | | ***Mean ± SD*** | ***p*** | ***d*** | **CI*d*** |
| A0 vs. | **A1** | 40.96 ± 18.36 vs 27.50 ± 12.81 | 0.198 | **0.8** | -1.444 – -0.235 |
|  | **A4** | 40.96 ± 18.36 vs 24.87 ± 16.14 | **0.034** | **0.9** | -1.528 – -0.322 |
|  | **A8** | 40.96 ± 18.36 vs 22.50 ± 9.132 | **0.002** | **1.3** | -1.890 – -0.645 |
|  | **A12** | 40.96 ± 18.36 vs 31.08 ± 13.99 | 0.926 | 0.6 | -1.181 – -0.023 |
| A1 vs. | **A4** | 27.50 ± 12.81 vs 24.87 ± 16.14 | >0.999 | 0.2 | -0.765 – 0.407 |
|  | **A8** | 27.50 ± 12.81 vs 22.50 ± 9.132 | >0.999 | 0.5 | -1.037 – 0.136 |
|  | **A12** | 27.50 ± 12.81 vs 31.08 ± 13.99 | >0.999 | 0.3 | -0.316 – 0.846 |
| A4 vs. | **A8** | 24.87 ± 16.14 vs 22.50 ± 9.132 | >0.999 | 0.2 | -0.754 – 0.392 |
|  | **A12** | 24.87 ± 16.14 vs 31.08 ± 13.99 | >0.999 | 0.4 | -0.168 – 0.988 |
| A8 vs. | **A12** | 22.50 ± 9.132 vs 31.08 ± 13.99 | 0.372 | 0.7 | 0.138 – 1.308 |
| Main effect: H(4) = 16.27, *p* = 0.0027** | | | | | |

1. **Time Spent in Top Zone**

| Exposure groups | | Descriptive statistics | | | |
| --- | --- | --- | --- | --- | --- |
|  | | ***Mean ± SD*** | ***p*** | ***d*** | **CI*d*** |
| A0 vs. | **A1** | 94.94 ± 53.59 vs 138.4 ± 61.98 | 0.268 | **0.8** | 0.149 – 1.348 |
|  | **A4** | 94.94 ± 53.59 vs 129.6 ± 92.21 | >0.999 | 0.5 | -0.120 – 1.039 |
|  | **A8** | 94.94 ± 53.59 vs 140.9 ± 70.50 | 0.168 | 0.7 | 0.144 – 1.315 |
|  | **A12** | 94.94 ± 53.59 vs 178.2 ± 67.21 | **0.0002** | **1.4** | 0.733 – 1.994 |
| A1 vs. | **A4** | 138.4 ± 61.98 vs 129.6 ± 92.21 | >0.999 | 0.1 | -0.696 – 0.474 |
|  | **A8** | 138.4 ± 61.98 vs 140.9 ± 70.50 | >0.999 | 0.04 | -0.541 – 0.616 |
|  | **A12** | 138.4 ± 61.98 vs 178.2 ± 67.21 | 0.449 | 0.6 | 0.019 – 1.204 |
| A4 vs. | **A8** | 129.6 ± 92.21 vs 140.9 ± 70.50 | >0.999 | 0.1 | -0.436 – 0.710 |
|  | **A12** | 129.6 ± 92.21 vs 178.2 ± 67.21 | **0.042** | 0.6 | 0.016 – 1.186 |
| A8 vs. | **A12** | 140.9 ± 70.50 vs 178.2 ± 67.21 | 0.544 | 0.5 | -0.038 – 1.115 |
| Main effect: *H*(4) = 19.73, *p* = 0.0006*** | | | | | |
