# Supplementary material for: The number of conspecific alarm substance donors notably influences the behavioural responses of zebrafish subjected to a traumatic stress procedure
Source: Fish Physiol Biochem. 2025 Feb 26;51(2):55. doi: 10.1007/s10695-025-01468-0 (PMC11865224; doi:10.1007/s10695-025-01468-0)
Supplement: Supplementary file 4 — Supplementary file4 (DOCX 38 KB) [file 10695_2025_1468_MOESM4_ESM.docx]

**The number of conspecific alarm substance donors notably influences the behavioural responses of zebrafish subjected to a traumatic stress procedure**

**Journal: Fish Physiology and Biochemistry**

**C van Staden^a^, K Finger-Baier^b^, D Weinshenker^c^, TL Botha^d^, L Brand^a^, D Wolmarans^a,^***

*^a^Centre of Excellence for Pharmaceutical Sciences, Department of Pharmacology, North-West University, 11 Hoffman Street, Potchefstroom, 2520, South Africa*

*^b^Department Genes - Circuits - Behavior, Max Planck Institute for Biological Intelligence, Martinsried, Germany*

*^c^Department of Human Genetics, Emory University School of Medicine, 615 Michael St., Whitehead 301, Atlanta, GA 30322, USA*

*^d^Department of Zoology, University of Johannesburg, Auckland Park, Johannesburg, 2006, South Africa*

Address correspondence to: De Wet Wolmarans, Center of Excellence for Pharmaceutical Sciences, Faculty of Health Sciences, North-West University, 11 Hoffman Street, Potchefstroom, South Africa.

Email: dewet.wolmarans@nwu.ac.za Telephone: +27 (0) 18 299 2230

**Table 4 – Descriptive statistics pertaining to behaviour of adult fish nOFT**

1. **Locomotor Activity**

| Exposure groups | | Descriptive statistics | | | |
| --- | --- | --- | --- | --- | --- |
|  | | ***Mean ± SD*** | ***p*** | ***d*** | **CI*d*** |
| A0 vs. | **A1** | 1880 ± 942.9 vs 1826 ± 602.5 | >0.999 | 0.1 | -0.646 – 0.511 |
|  | **A4** | 1880 ± 942.9 vs 1506 ± 801.1 | >0.999 | 0.4 | -1.004 – 0.154 |
|  | **A8** | 1880 ± 942.9 vs 1936 ± 502.7 | >0.999 | 0.1 | -0.493 – 0.639 |
|  | **A12** | 1880 ± 942.9 vs 1857 ± 915.8 | >0.999 | 0 | -0.591 – 0.541 |
| A1 vs. | **A4** | 1826 ± 602.5 vs 1506 ± 801.1 | 0.850 | 0.5 | -1.040 – 0.144 |
|  | **A8** | 1826 ± 602.5 vs 1936 ± 502.7 | >0.999 | 0.2 | -0.383 – 0.777 |
|  | **A12** | 1826 ± 602.5 vs 1857 ± 915.8 | >0.999 | 0 | -0.540 – 0.617 |
| A4 vs. | **A8** | 1506 ± 801.1 vs 1936 ± 502.7 | 0.306 | 0.6 | 0.056 – 1.230 |
|  | **A12** | 1506 ± 801.1 vs 1857 ± 915.8 | >0.999 | 0.4 | -0.173 – 0.983 |
| A8 vs. | **A12** | 1936 ± 502.7 vs 1857 ± 915.8 | >0.999 | 0.1 | -0.673 – 0.460 |
| Main effect: *H*(4) = 5.32, *p* = 0.256 | | | | | |

1. **Time Spent Freezing**

| Exposure groups | | Descriptive statistics | | | |
| --- | --- | --- | --- | --- | --- |
|  | | ***Mean ± SD*** | ***p*** | ***d*** | **CI*d*** |
| A0 vs. | **A1** | 62.93 ± 69.98 vs 28.89 ± 59.67 | **0.034** | 0.5 | -1.107 – 0.070 |
|  | **A4** | 62.93 ± 69.98 vs 82.78 ± 92.38 | >0.999 | 0.2 | -0.332 – 0.816 |
|  | **A8** | 62.93 ± 69.98 vs 20.34 ± 50.31 | **0.024** | 0.7 | -1.279 – -0.112 |
|  | **A12** | 62.93 ± 69.98 vs 52.55 ± 77.01 | >0.999 | 0.1 | -0.707 – 0.426 |
| A1 vs. | **A4** | 28.89 ± 59.67 vs 82.78 ± 92.38 | **0.008** | 0.7 | 0.084 – 1.288 |
|  | **A8** | 28.89 ± 59.67 vs 20.34 ± 50.31 | >0.999 | 0.2 | -0.734 – 0.425 |
|  | **A12** | 28.89 ± 59.67 vs 52.55 ± 77.01 | 0.489 | 0.3 | -0.243 – 0.923 |
| A4 vs. | **A8** | 82.78 ± 92.38 vs 20.34 ± 50.31 | **0.006** | **0.8** | -1.438 – -0.242 |
|  | **A12** | 82.78 ± 92.38 vs 52.55 ± 77.01 | >0.999 | 0.4 | -0.931 – 0.222 |
| A8 vs. | **A12** | 20.34 ± 50.31 vs 52.55 ± 77.01 | 0.403 | 0.5 | -0.082 – 1.067 |
| Main effect: *H*(4) = 20.79, *p* = 0.0003*** | | | | | |

1. **Total Dark Time**

| Exposure groups | | Descriptive statistics | | | |
| --- | --- | --- | --- | --- | --- |
|  | | ***Mean ± SD*** | ***p*** | ***d*** | **CI*d*** |
| A0 vs. | **A1** | 109.2 ± 97.32 vs 52.76 ± 104.6 | 0.136 | 0.6 | -1.147 – 0.033 |
|  | **A4** | 109.2 ± 97.32 vs 129.5 ± 130.1 | >0.999 | 0.2 | -0.397 – 0.749 |
|  | **A8** | 109.2 ± 97.32 vs 39.89 ± 84.79 | **0.017** | **0.8** | -1.342 – -0.169 |
|  | **A12** | 109.2 ± 97.32 vs 104.9 ± 114.0 | >0.999 | 0 | -0.606 – 0.526 |
| A1 vs. | **A4** | 52.76 ± 104.6 vs 129.5 ± 130.1 | 0.075 | 0.6 | 0.044 – 1.245 |
|  | **A8** | 52.76 ± 104.6 vs 39.89 ± 84.79 | >0.999 | 0.1 | -0.714 – 0.444 |
|  | **A12** | 52.76 ± 104.6 vs 104.9 ± 114.0 | 0.863 | 0.5 | -0.114 – 1.060 |
| A4 vs. | **A8** | 129.5 ± 130.1 vs 39.89 ± 84.79 | **0.008** | **0.8** | -1.411 – -0.219 |
|  | **A12** | 129.5 ± 130.1 vs104.9 ± 114.0 | >0.999 | 0.2 | -0.773 – 0.374 |
| A8 vs. | **A12** | 39.89 ± 84.79 vs 104.9 ± 114.0 | 0.175 | 0.6 | 0.063 – 1.225 |
| Main effect: *H*(4) = 18.00, *p* = 0.0012** | | | | | |

1. **Total Border Time**

| Exposure groups | | Descriptive statistics | | | |
| --- | --- | --- | --- | --- | --- |
|  | | ***Mean ± SD*** | ***p*** | ***d*** | **CI*d*** |
| A0 vs. | **A1** | 25.64 ± 23.24 vs 31.82 ± 23.05 | >0.999 | 0.3 | -0.316 – 0.847 |
|  | **A4** | 25.64 ± 23.24 vs 26.30 ± 38.04 | >0.999 | 0 | -0.551 – 0.593 |
|  | **A8** | 25.64 ± 23.24 vs 26.61 ± 15.61 | >0.999 | 0 | -0.517 – 0.615 |
|  | **A12** | 25.64 ± 23.24 vs 19.41 ± 14.14 | >0.999 | 0.3 | -0.892 – 0.247 |
| A1 vs. | **A4** | 31.82 ± 23.05 vs 26.30 ± 38.04 | 0.833 | 0.2 | -0.759 – 0.421 |
|  | **A8** | 31.82 ± 23.05 vs 26.61 ± 15.61 | >0.999 | 0.3 | -0.846 – 0.316 |
|  | **A12** | 31.82 ± 23.05 vs 19.41 ± 14.14 | 0.923 | 0.7 | -1.247 – -0.058 |
| A4 vs. | **A8** | 26.30 ± 38.04 vs 26.61 ± 15.61 | >0.999 | 0 | -0.561 – 0.583 |
|  | **A12** | 26.30 ± 38.04 vs 19.41 ± 14.14 | >0.999 | 0.2 | -0.815 – 0.333 |
| A8 vs. | **A12** | 26.61 ± 15.61 vs 19.41 ± 14.14 | >0.999 | 0.5 | -1.056 – 0.093 |
| Main effect: *H*(4) = 5.07, *p* = 0.281 | | | | | |

1. **Total OF Time**

| Exposure groups | | | Descriptive statistics | | | |
| --- | --- | --- | --- | --- | --- | --- |
|  | | | ***Mean ± SD*** | ***p*** | ***d*** | **CI*d*** |
| A0 vs. | **A1** | 120.8 ± 79.15 vs 150.1 ± 85.12 | | >0.999 | 0.4 | -0.229 – 0.938 |
|  | **A4** | 120.8 ± 79.15 vs 78.18 ± 60.93 | | 0.610 | 0.6 | -1.184 – -0.013 |
|  | **A8** | 120.8 ± 79.15 vs 149.3 ± 85.06 | | >0.999 | 0.3 | -0.225 – 0.916 |
|  | **A12** | 120.8 ± 79.15 vs 94.22 ± 73.98 | | >0.999 | 0.3 | -0.915 – 0.225 |
| A1 vs. | **A4** | 150.1 ± 85.12 vs 78.18 ± 60.93 | | **0.024** | **1.0** | -1.589 – -0.350 |
|  | **A8** | 150.1 ± 85.12 vs 149.3 ± 85.06 | | >0.999 | 0.01 | -0.587 – 0.570 |
|  | **A12** | 150.1 ± 85.12 vs 94.22 ± 73.98 | | 0.167 | 0.7 | -1.295 – -0.102 |
| A4 vs. | **A8** | 78.18 ± 60.93 vs 149.3 ± 85.06 | | **0.026** | **1.0** | 0.348 – 1.558 |
|  | **A12** | 78.18 ± 60.93 vs 94.22 ± 73.98 | | >0.999 | 0.2 | -0.339 – 0.809 |
| A8 vs. | **A12** | 149.3 ± 85.06 vs 94.22 ± 73.98 | | 0.186 | 0.7 | -1.271 – -0.105 |
| Main effect: *H*(4) = 15.02, *p* = 0.005** | | | | | | |

1. **Total Time at Aggressor**

| Exposure groups | | Descriptive statistics | | | |
| --- | --- | --- | --- | --- | --- |
|  | | ***Mean ± SD*** | ***p*** | ***d*** | **CI*d*** |
| A0 vs. | **A1** | 16.61 ± 31.39 vs 46.74 ± 63.45 | >0.999 | 0.6 | 0.015 – 1.199 |
|  | **A4** | 16.61 ± 31.39 vs 53.19 ± 68.96 | >0.999 | 0.7 | 0.095 – 1.273 |
|  | **A8** | 16.61 ± 31.39 vs 64.11 ± 68.45 | 0.141 | **0.9** | 0.293 – 1.482 |
|  | **A12** | 16.61 ± 31.39 vs 70.23 ± 72.50 | 0.160 | **1.0** | 0.356 – 1.554 |
| A1 vs. | **A4** | 46.74 ± 63.45 vs 53.19 ± 68.96 | >0.999 | 0.1 | -0.488 – 0.682 |
|  | **A8** | 46.74 ± 63.45 vs 64.11 ± 68.45 | >0.999 | 0.3 | -0.320 – 0.842 |
|  | **A12** | 46.74 ± 63.45 vs 70.23 ± 72.50 | >0.999 | 0.3 | -0.241 – 0.925 |
| A4 vs. | **A8** | 53.19 ± 68.96 vs 64.11 ± 68.45 | >0.999 | 0.2 | -0.415 – 0.731 |
|  | **A12** | 53.19 ± 68.96 vs 70.23 ± 72.50 | >0.999 | 0.2 | -0.335 – 0.813 |
| A8 vs. | **A12** | 64.11 ± 68.45 vs 70.23 ± 72.50 | >0.999 | 0.1 | -0.480 – 0.652 |
| Main effect: *H*(4) = 8.09, *p* = 0.089 | | | | | |

Descriptive statistics of **Total Dark Entries**

| Exposure groups | | Descriptive statistics | | | | |  |
| --- | --- | --- | --- | --- | --- | --- | --- |
|  | | ***Mean ± SD*** | ***p*** | ***d*** | **CI*d*** |  |  |
| A0 vs. | **A1** | 9.167 ± 7.597 vs 4.091 ± 7.533 | **0.049** | 0.7 | -1.262 – -0.072 |  |  |
|  | **A4** | 9.167 ± 7.597 vs 4.435 ± 5.177 | >0.999 | 0.7 | -1.312 – -0.130 |  |  |
|  | **A8** | 9.167 ± 7.597 vs 2.708 ± 8.180 | **0.0004** | **0.8** | -1.404 – -0.224 |  |  |
|  | **A12** | 9.167 ± 7.597 vs 5.375 ± 9.093 | 0.245 | 0.5 | -1.023 – 0.123 |  |  |
| A1 vs. | **A4** | 4.091 ± 7.533 vs 4.435 ± 5.177 | >0.999 | 0.1 | -0.531 – 0.638 |  |  |
|  | **A8** | 4.091 ± 7.533 vs 2.708 ± 8.180 | >0.999 | 0.2 | -0.754 – 0.405 |  |  |
|  | **A12** | 4.091 ± 7.533 vs 5.375 ± 9.093 | >0.999 | 0.2 | -0.427 – 0.732 |  |  |
| A4 vs. | **A8** | 4.435 ± 5.177 vs 2.708 ± 8.180 | 0.055 | 0.3 | -0.824 – 0.325 |  |  |
|  | **A12** | 4.435 ± 5.177 vs 5.375 ± 9.093 | >0.999 | 0.1 | -0.447 – 0.698 |  |  |
| A8 vs. | **A12** | 2.708 ± 8.180 vs 5.375 ± 9.093 | 0.615 | 0.3 | -0.263 – 0.876 |  |  |
| Main effect: *H*(4) = 19.31, *p* = 0.0007*** | | | | | | | |

Descriptive statistics of **Total Entries at Aggressor**

| Exposure groups | | Descriptive statistics | | | | |  |
| --- | --- | --- | --- | --- | --- | --- | --- |
|  | | ***Mean ± SD*** | ***p*** | ***d*** | **CI*d*** |  |  |
| A0 vs. | **A1** | 11.96 ± 17.41 vs 18.09 ± 17.16 | >0.999 | 0.4 | -0.230 – 0.936 |  |  |
|  | **A4** | 11.96 ± 17.41 vs 20.17 ± 20.06 | >0.999 | 0.4 | -0.143 – 1.015 |  |  |
|  | **A8** | 11.96 ± 17.41 vs 22.96 ± 18.98 | 0.617 | 0.6 | 0.022 – 1.180 |  |  |
|  | **A12** | 11.96 ± 17.41 vs 29.04 ± 25.62 | 0.260 | **0.8** | 0.188 – 1.364 |  |  |
| A1 vs. | **A4** | 18.09 ± 17.16 vs 20.17 ± 20.06 | >0.999 | 0.1 | -0.474 – 0.696 |  |  |
|  | **A8** | 18.09 ± 17.16 vs 22.96 ± 18.98 | >0.999 | 0.3 | -0.314 – 0.848 |  |  |
|  | **A12** | 18.09 ± 17.16 vs 29.04 ± 25.62 | >0.999 | 0.5 | -0.093 – 1.083 |  |  |
| A4 vs. | **A8** | 20.17 ± 20.06 vs 22.96 ± 18.98 | >0.999 | 0.1 | -0.431 – 0.715 |  |  |
|  | **A12** | 20.17 ± 20.06 vs 29.04 ± 25.62 | >0.999 | 0.4 | -0.195 – 0.960 |  |  |
| A8 vs. | **A12** | 22.96 ± 18.98 vs 29.04 ± 25.62 | >0.999 | 0.3 | -0.300 – 0.837 |  |  |
| Main effect: *H*(4) = 6.12, *p* = 0.190 | | | | | | | |
